# Relevance of Copper and Organic Cation Transporters in the Activity and Transport Mechanisms of an Anticancer Cyclometallated Gold(III) Compound in Comparison to Cisplatin

**DOI:** 10.3389/fchem.2018.00377

**Published:** 2018-09-04

**Authors:** Sarah Spreckelmeyer, Margot van der Zee, Benoît Bertrand, Ewen Bodio, Stefan Stürup, Angela Casini

**Affiliations:** ^1^Department Pharmacokinetics, Toxicology and Targeting, Groningen Research Institute of Pharmacy, University of Groningen, Groningen, Netherlands; ^2^Medicinal Inorganic Chemistry Group, Department of Chemistry, University of British Columbia, Vancouver, BC, Canada; ^3^ICMUB UMR6302, CNRS, Université Bourgogne Franche-Comté, Dijon, France; ^4^Department of Pharmacy, University of Copenhagen, Copenhagen, Denmark; ^5^School of Chemistry, Cardiff University, Cardiff, United Kingdom

**Keywords:** organometallic gold compounds, cisplatin, cancer, drug transporters, organic cation transporters, copper transporters, ICP-MS

## Abstract

The molecular mechanisms of toxicity and cellular transport of anticancer metallodrugs, including platinum-based agents, have not yet been fully elucidated. The aim of our study was to investigate the relevance of copper transporters (CTR1 and ATP7A/B), organic cation transporters (OCT2) and the multidrug and toxin extrusion proteins (MATE) in the intracellular accumulation of a novel organometallic cytotoxic Au(III) compound in cancer cells in comparison to cisplatin. Specifically, the synthesis and characterization of the gold complex [Au(py^b^-H)(PPh_2_Ar)Cl]PF_6_ (PPh_2_Ar = 3-[4-(diphenylphosphino)phenyl]-7-methoxy-2H-chromen-2-one] (**1**), featuring a coumarin ligand endowed with “smart” fluorescence properties, have been achieved. Initially, the cytotoxic effects of both cisplatin and **1** were studied in a small panel of human cancer cells, and against a non-tumorigenic cell line *in vitro*. Thus, the human ovarian cancer cell line A2780 and its cisplatin resistant variant A2780cisR, were selected, being most sensitive to the treatment of the gold complex. Co-incubation of the metallodrugs with CuCl_2_ (a CTR1 substrate) increased the cytotoxic effects of both the Au(III) complex and cisplatin; while co-incubation with cimetidine (inhibitor of OCT2 and MATE) showed some effect only after 72 h incubation. ICP-MS (Inductively Coupled Plasma Mass Spectrometry) analysis of the cell extracts showed that co-incubation with CuCl_2_ increases Au and Cu accumulation in both cancer cell lines, in accordance with the enhanced antiproliferative effects. Conversely, for cisplatin, no increase in Pt content could be observed in both cell lines after co-incubation with either CuCl_2_ or cimetidine, excluding the involvement of CTR1, OCT2, and MATE in drug accumulation and overall anticancer effects. This result, together with the evidence for increased Cu content in A2780 cells after cisplatin co-treatment with CuCl_2_, suggests that copper accumulation is the reason for the observed enhanced anticancer effects in this cell line. Moreover, metal uptake studies in the same cell lines indicate that both **1** and cisplatin are not transported intracellularly by CTR1 and OCT2. Finally, preliminary fluorescence microscopy studies enabled the visualization of the sub-cellular distribution of the gold compound in A2780 cells, suggesting accumulation in specific cytosolic components/organelles.

## Introduction

**Figure 1:**
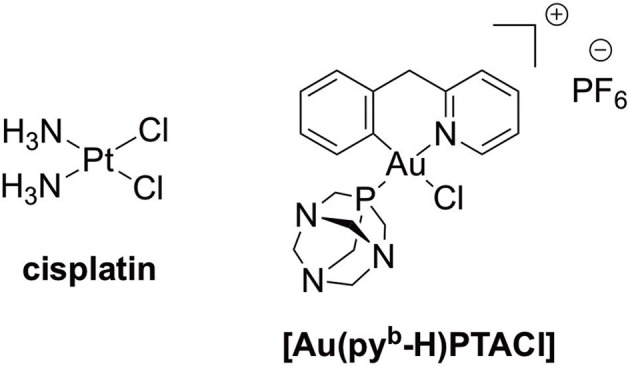
The anticancer Pt(II) drug, cisplatin, and cyclometallated [Au(py^b^-H)PTACl] (py^b^-H = C^∧^N cyclometallated 2-benzylpyridine, PTA = 1,3,5-triazaphosphaadamantane).

In the field of anticancer metallodrugs, platinum(II) compounds (cisplatin (Figure 1), carboplatin, oxaliplatin, and nedaplatin) are still the gold-standard treatment for numerous types of cancers,(Desoize and Madoulet, 2002) mostly applied in combination with other chemotherapeutics. However, their use is limited due to intrinsic or acquired resistance and severe side-effects, such as nephrotoxicity in the case of cisplatin. Consequently, a panel of non-platinum metallodrugs was designed as anticancer agents to achieve improved therapeutics. Many of them are in preclinical studies, but only a few reached clinical trials and are still far from being marketed (Ang et al., 2011; Jaouen et al., 2015; Meier-Menches et al., 2018). In general, the differential activity/toxicity profiles of anticancer drugs in different organs, such as the nephrotoxicity of cisplatin, may be related to different drug accumulation in the cells of these tissues. It is worth mentioning that higher drug accumulation in a particular cell type might be caused by either higher uptake or lower efflux(Fletcher et al., 2010; Russel, 2010), which emphasizes the need for mechanistic information on the drug transport mechanisms involved and possible interactions with membrane transporters. However, even for the well-established anticancer platinum drugs, knowledge about their mechanism of accumulation in cancer cells, as well as in healthy tissues, is incomplete and often contradictory. Specifically, the hOCT2 (human organic cation transporter 2) and hCTR1 (human copper transporter 1) have been postulated to be involved in the uptake of cisplatin, whereas the multidrug and toxic extrusion protein (MATE) and ATP7A/B seem to be involved in the drug's efflux, as recently reviewed by Spreckelmeyer et al. (2014) and others (Hall et al., 2008; Howell et al., 2010). It should be noted that while hCTR1 is responsible for the cellular uptake of Cu^+^ ions, the ATP7A and ATP7B are pumps known to be involved in the efflux of copper ions. MATEs are part of the organic cation homeostasis, belonging to the SLC47 family. In fact, MATEs act as H^+^/organic cation antiporters, transporting protons from the extracellular side to the cytoplasm in concomitance with organic cations export to the lumen of the proximal tubule. Several studies have confirmed cisplatin transport by MATEs (Ciarimboli, 2014).

Recently, Natile, Arnesano, and co-workers investigated the binding of platinum(II) complexes to the Met-rich domain of hCTR1 by several methods including mass spectrometry and NMR spectroscopy (Arnesano et al., 2007; Alessia et al., 2018). According to the obtained results, cisplatin reacts with the copper binding domain forming adducts where all the original ligands of Pt(II) are substituted by the S-donor groups of Met side-chains. According to these observations, cisplatin would be actually sequestered by hCTR1 and not transported intracellularly. Furthermore, regulation of the expression of these membrane transporters upon metallodrug administration can also occur and its effects on cisplatin treatment should be considered (Holzer and Howell, 2006). Recently, Howell and coworkers reported on the use of the CRISPR-Cas9 technique to individually knock out the human copper transporters CTR1 and CTR2 and the copper chaperones ATOX1 and CCS in human cancer cells (Bompiani et al., 2016). The obtained results showed that loss of such transporters does not produce a change in cisplatin sensitivity.

Among new generation metal-based complexes, gold(I) and gold(III) compounds raised interest in the last years showing promising anticancer properties *in vitro*, which prompted the investigation of their molecular mechanisms of biological action (Ott, 2009; Bertrand and Casini, 2014). As a representative example, the gold(I) compound auranofin ([Au(I)(2,3,4,6-tetra-O-acetyl-1-(thio-κS)-β-D-glucopyranosato)(triethylphosphine)]) is a potent cytotoxic agent *in vitro* and is currently in clinical trials (Nobili et al., 2010). Among the various families of gold compounds tested for their anticancer effects in the last decade, a variety of organometallic gold(I/III) compounds appeared to be particularly promising (Muenzner et al., 2015; Sigel et al., 2018). Indeed, they feature potent anticancer activity *in vitro*, and are endowed with improved stability in physiological environment with respect to classical coordination complexes. In this context, our group has designed new gold(III) cyclometallated compounds featuring both bidentate C^∧^N- and tridentate C^∧^N^∧^N-, C^∧^N^∧^C-, and N^∧^C^∧^N-donor ligands, with either five- or six-membered C,N rings.(Bertrand and Casini, 2014; Fung et al., 2017; Jurgens and Casini, 2017; Jurgens et al., 2017a; Jürgens et al., 2017b; Williams et al., 2017; Bertrand et al., 2018) For example, a series of C^∧^N gold(III) complexes of general formula [Au(py^b^-H)X_2_] (py^b^-H = C^∧^N cyclometallated 2-benzylpyridine) has been developed by us showing interesting antiproliferative effects in a panel of human cancer cells *in vitro* (Bertrand et al., 2015).

While investigation of the possible pharmacological targets for this family of organometallic gold(III) complexes has led to the identification of a number of cancer-related proteins, including the seleno-enzyme thioredoxin reductase, (Gabbiani et al., 2011) and zinc finger proteins involved in DNA repair mechanisms, (Wenzel et al., 2018) among others, very scarce information is available concerning the drug transport mechanisms in cancer cells. As far as we are aware, only one *in vitro* study has been published evaluating the role of hOCTs, hCTR1, and of endocytotic processes in the uptake of a cytotoxic gold(I) NHC (N-heterocyclic carbene) complex in cancer cells (Kaps et al., 2012). The results suggest that cellular uptake for all tested gold complexes occurs mainly *via* the OCT transporters, but in one case also *via* hCTR1. In addition, some of the compounds were also internalized *via* the Na^+^/K^+^-dependent endocytosis (Kaps et al., 2012). In fact, while in general endocytosis is the vesicle-mediated process used by all cells to internalize extracellular molecules, some endocytotic processes depend on a sodium gradient and can be slowed down by adding ouabaine, an inhibitor of the Na^+^/K^+^ pump.

More recently, we reported on the toxic effects and accumulation mechanisms of another C^∧^N gold(III) complex–[Au(py^b^-H)(PTA)Cl] (PTA = 1,3,5-triazaphosphaadamantane) (Figure 1)–in healthy rat kidneys *ex vivo*, using the Precision Cut Tissue Slices (PCTS) method (Spreckelmeyer et al., 2017). Interestingly, the obtained results seem to exclude OCTs-related uptake mechanisms as well as MATE efflux pathways for this gold(III) compound in kidneys.

**Figure 2:**
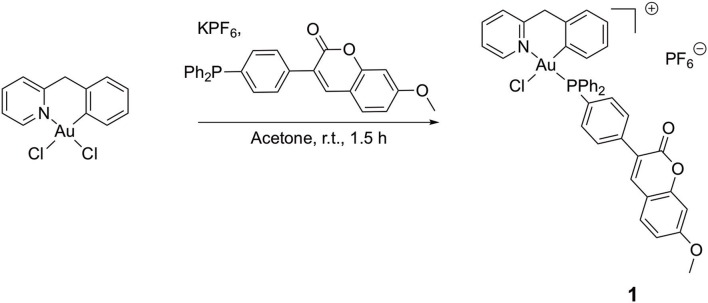
Synthesis of compound [Au(py^b^-H)(PPh_2_Ar)Cl]PF_6_ (PPh_2_Ar = 3-[4 (diphenylphosphino)phenyl]-7-methoxy-2H-chromen-2-one] **1**.

Here, we report on the synthesis and characterization of a novel cyclometallated C^∧^N gold(III) compound (Figure 2) [Au(py^b^-H)(PPh_2_Ar)Cl]PF_6_ (PPh_2_Ar = 3-[4-(diphenylphosphino)phenyl]-7-methoxy-2H-chromen-2-one] (**1**) featuring a coumarin ligand endowed with “smart” fluorescence properties for imaging in cells by fluorescence microscopy (Hanthorn et al., 2012; Ali et al., 2015; Dondaine et al., 2016). The smart character of this probe involves a dramatic drop of the fluorescence when the metal is released, allowing a reliable tracking of the complex: if strong fluorescence is observed it means that the “P-Au” bond is intact. The evaluation of the antiproliferative effects of the Au(III) complex compared to cisplatin was performed using a panel of human cancer cell lines as well as non-cancerous cells *in vitro*. Moreover, the involvement of different transporters (CTR1, OCT2, MATE, ATP7A/B) in the antiproliferative activity of the gold compound was studied *via* transporter inhibition experiments in comparison to cisplatin in two human ovarian cancer cell lines, one sensitive (A2780) and one resistant to cisplatin (A2780cisR). Furthermore, the accumulation and uptake mechanisms of the compounds were studied in the same cells, evaluating the cellular metal content by Inductively Coupled Plasma Mass Spectrometry (ICP-MS). Preliminary fluorescence microscopy data allowed investigation of the sub-cellular localization of complex **1** in cancer cells.

## Materials and methods

### General remarks

All reactions were carried out under an atmosphere of purified argon using Schlenk techniques. Solvents were dried and distilled under argon before use. Cisplatin was purchased at Sigma, while the precursor [Au(py^b^-H)Cl_2_] and the coumarin-phosphine have been synthesized according to literature procedure (Cinellu et al., 1995; Hanthorn et al., 2012). The identity and purity (≥95%) of the gold complexes under investigation were unambiguously established using high-resolution mass spectrometry and NMR. All other reagents were commercially available and used as received. All the analyses were performed at the “Plateforme d'Analyses Chimiques et de Synthèse Moléculaire de l'Université de Bourgogne.” Exact masses of the synthesized complexes were obtained on a Thermo LTQ Orbitrap XL. ^1^H- (300.13, 500.13, or 600.23 MHz), ^13^C- (125.77 or 150.90 MHz) and ^31^P- (121.49, 202.45, or 242.94 MHz) NMR spectra were recorded on Bruker 300 Avance III, 500 Avance III or 600 Avance II spectrometers. Chemical shifts are quoted in ppm (δ) relative to TMS (^1^H and ^13^C) using the residual protonated solvent (^1^H) or the deuterated solvent (^13^C) as internal standards. 85% H_3_PO_4_ (^31^P) was used as an external standard. Infrared spectra were recorded on a Bruker Vector 22 FT-IR spectrophotometer (Golden Gate ATR) and far infrared spectra were recorded on a Bruker Vertex 70v FT-IR spectrophotometer (Diamant ATR).

### Synthesis of [Au(py^b^-H)(PPh_2_Ar)Cl].PF_6_ (1)

A round-bottom flask was charged with the precursor [Au(py^b^-H)Cl_2_] (50 mg, 0.115 mmol, 1 eq.), KPF_6_ (106 mg, 0.573 mmol, 5 eq.) and 3-[4-(diphenylphosphino)phenyl]-7-methoxy-2H-chromen-2-one (PPh_2_Ar) (50 mg, 0.115 mmol, 1 eq.) in suspension into 5 mL of distilled acetone under argon atmosphere. The gold complex precursor was solubilized after few minutes. The reaction was maintained at room temperature for 1.5 h; afterward, 10 mL of dichloromethane were added, and the yellow solution was filtrated through Celite^®^ and concentrated under vacuum. The pure product was obtained after precipitation from a dichloromethane/pentane mixture as a yellow powder (91 mg, 0.092 mmol, 80 % yield).

^1^H NMR (Acetone-d6, 500.13 MHz, 298 K): 3.97 (s, 3 H, OCH_3_), 4.49 (d, 1 H, ^2^*J*_H−H_ = 15.6 Hz, CH_2−PyrBz_), 5.05 (d, 1 H, ^2^*J*_H−H_ = 15.6 Hz, CH_2−PyrBz_), 6.52 (dt, 1 H, ^3^*J*_H−H_ = 8.5 Hz, ^4^*J*_H−H_ = 1.5 Hz, H^5^′), 6.88 (dd, 1 H, ^3^*J*_H−H_ = 7.5 Hz, ^4^*J*_H−H_ = 3.0 Hz, H^6^′), 6.98 (d, 1 H, ^4^*J*_H−H_ = 2.5 Hz, H^D^), 7.00 (dd, ^3^*J*_H−H_ = 8.5 Hz, ^4^*J*_H−H_ = 2.5 Hz, H^C^), 7.03 (dt, ^3^*J*_H−H_ = 8.5 Hz, ^4^*J*_H−H_ = 0.5 Hz, H^4^′), 7.33 (dd, 1 H, ^3^*J*_H−H_ = 8.5 Hz, ^4^*J*_H−H_ = 1.5 Hz, H^3^′), 7.60–7.66 (m, 4 H, H_ortho−Ph_), 7.70 (d, ^3^*J*_H−H_ = 8.5 Hz, H^B^), 7.73–7.79 (m, 2 H, H_ortho−pC6H4_), 7.81 (t, ^3^*J*_H−H_ = 8.5 Hz, H^5^), 7.89–8.01 (m, 8 H, H_meta/para−Ph_ + H_meta−pC6H4_), 8.06 (d, 1 H, ^3^*J*_H−H_ = 8.5 Hz, H^3^), 8.27 (s, 1 H, H^A^), 8.31 (dt, 1 H, ^3^*J*_H−H_ = 8.5 Hz, ^4^*J*_H−H_ = 1.5 Hz, H^4^), 9.25 (broad s, 1 H, H^6^).

^13^C(^1^H) NMR (Acetone-d6, 125.77 MHz, 300 K): 47.9 (s, *C*H_2−PyrBz_), 56.5 (s, O -*C*H_3_), 101.2 (s, *C*H^D^), 113.8 (s, *C*H^C^), 114.0 (s, *C*_quat−coum_), 122.9 (s, *C*_quat−p−C6H4_), 124.0 (d, ^1^*J*_P−C_ = 84.3 Hz, *C*_ipso−Ph_), 124.5 (d, ^1^*J*_P−C_ = 83.0 Hz, *C*_ipso−Ph_), 124.8 (d, ^1^*J*_P−C_ = 70.4 Hz, *C*_ipso−p−C6H4_), 125.4 (d, ^4^*J*_P−C_ = 3.8 Hz, *C*H^5^), 127.3 (d, ^4^*J*_P−C_ = 3.8 Hz, *C*H^3^), 128.8 (s, *C*H^4^′), 128.9 (d, ^4^*J*_P−C_ = 2.5 Hz, *C*H^5^′),129.8 (s, *C*H_para−Ph_), 129.9 (s, *C*H^3^′), 130.2 (d, ^2^*J*_P−C_ = 10.1 Hz, *C*H_ortho−Ph_), 130.3 (d, ^2^*J*_P−C_ = 10.1 Hz, *C*H_ortho−Ph_), 131.0 (s, *C*H^B^), 133.7 (d, ^3^*J*_P−C_ = 7.5 Hz, *C*H^6^′), 134.5 (d, ^3^*J*_P−C_ = 2.5 Hz, *C*H_ortho−p−C6H4_), 134.6 (s + d, ^3^*J*_P−C_ = 2.5 Hz, *C*_quat−Bz_ + *C*H_ortho−p−C6H4_), 136.1 (s, *C*H_meta−Ph_), 136.2 (s, *C*H_meta−Ph_ + *C*H_meta−p−C6H4_), 136.4 (s, *C*H_meta−p−C6H4_), 141.6 (d, ^2^*J*_P−C_ = 2.5 Hz, *C*-Au), 143.1 (s, *C*H^A^), 144.2 (s, *C*H^4^), 150.8 (s, *C*_quat−coum_), 152.4 (s, *C*H^6^), 156.8 (s, *C*_quat−pyr_), 157.9 (s, *C*_quat−coum_), 160.4 (s, *C*_quat−coum_), 164.6 (s, *C*_quat−coum_).

^31^P(^1^H) NMR (Acetone-d6, 202.45 MHz, 300 K): 31.5 (s, 1 P, PPh_3_-Coum),−144.2 (h, 1 P, PF_6_).

ESI-MS (DMSO-MeOH), *positive mode exact mass* for [C_40_H_31_NO_3_PAuCl]^+^ (836.13901): measured *m/z* 836.13656 [M-PF_6_]^+^.

IR (ATR & FIR, cm^−1^): 1725, 1613, 1569, 1437, 1362, 1025, 836, 751, 311, 229.

Anal. Calc. for C_40_H_31_NO_3_P_2_F_6_AuCl: C, 48.92, H, 3.18, N, 1.43%. Found: C, 48.40, H, 2.70, N, 1.52%.


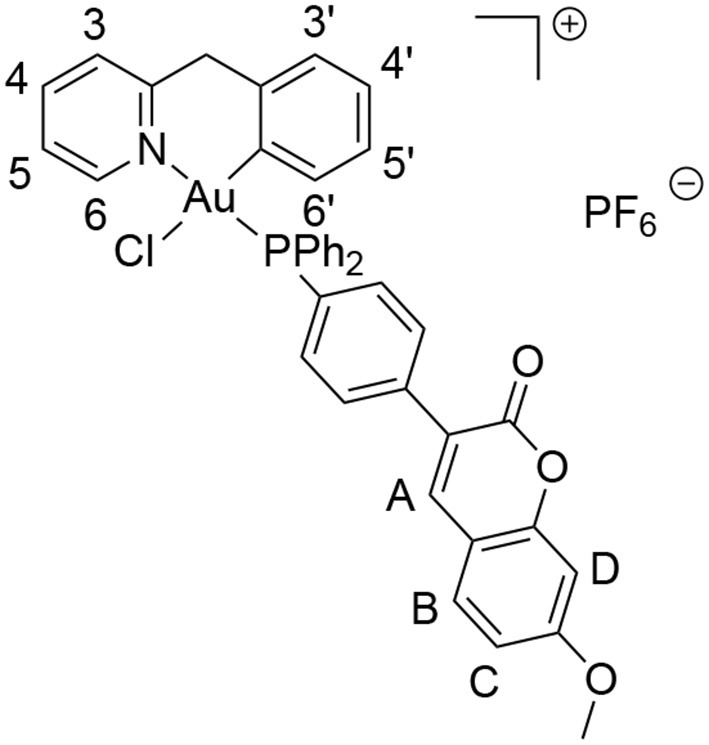


### Photophysical characterization

UV-Visible absorption spectra were recorded on a JASCO V630BIO spectrometer. The steady-state fluorescence emission spectra were obtained by using a JASCO FP8560 spectrofluorimeter instrument. All fluorescence spectra were corrected for instrument response. The fluorescence quantum yields (Φ_F_) were calculated from equation:

$$\frac{\Phi_{F}}{\Phi_{FR}}=\frac{n^{2}}{n^{2}{}_{R}}\times \frac{\int_{0}^{\infty }I_{F}(\lambda_{E},\lambda_{F})d\lambda_{F}}{\int_{0}^{\infty }I_{FR}(\lambda_{E},\lambda_{F})d\lambda_{F}}\times \frac{1-10^{-A_{R}(\lambda_{E})}}{1-10^{-A(\lambda_{E})}}$$

Φ_F_ and Φ_FR_ are fluorescence quantum yields of the compound and the reference respectively. A(λ_E_) and A_R_(λ_E_) are the absorbance at the excitation wavelength, and n is the refractive index of the medium. I_F_ and I_FR_ are fluorescent intensities of the compound and the reference respectively. 9,10-diphenylanthacene (Φ_F_ = 0.97 in cyclohexane) was used as standard (Brouwer Albert, 2011). In all Φ_F_ determinations, correction for the solvent refractive index (η) was applied.

### Cell viability assay

The human breast cancer cell line MCF7, human lung cancer cell line A549, human colon cancer cell line HCT116 p53 wild-type (+/+) (obtained from the European Centre of Cell Cultures ECACC, Salisbury, UK) and a p53 null variant of HCT116 (–/–) (kindly provided by Dr. G. Hartleben, University of Groningen), as well as non-cancerous human embryonic kidney cells HEK-293T (provided by Dr. M. P. Rigobello, University of Padova, Italy) were cultured in DMEM (Dulbecco's Modified Eagle Medium). Human ovarian sensitive (A2780) and cisplatin resistant (A2780cisR) cells were grown in Roswell Park Memorial Institute (RPMI) 1,640 medium. Both media contained GlutaMax supplemented with 10% FBS and 1% penicillin/streptomycin (all from Invitrogen). Cells were incubated at 37°C in a humidified atmosphere of 95% of air and 5% CO_2_ in an incubator (Heraeus, Germany). In the case of the A2780 and A2780cisR cells, the medium was also supplemented with 2 mM L-glutamine. To maintain resistance, cisplatin-resistant A2780cisR cells were treated with 1 μM cisplatin between every third passage.

For evaluation of toxicity, cells were seeded in 96-well plates (Costar, Integra Biosciences, Cambridge, MA) at a concentration of 10.000 cells/well (A2780, A2780cisR, MCF-7, HEK-293T) or 6,000 cells/well (HCT116 p53 +/+, HCT116 p53 –/–, A549) and grown for 24 h in the appropriate medium mentioned above. Solutions of the compounds were prepared by diluting a freshly prepared stock solution (10^−2^ M in DMSO for **1** or 10^−3^ M in aqueous solution for cisplatin) of the corresponding compound in media (RPMI or DMEM for the A2780 or A549, MCF-7, HCT116 p53+/+, HCT116 p53–/–, and HEK-293T).

Afterwards, 200 μL of these dilutions of the compounds were added to the wells to obtain a final concentration ranging from 0 to 120 μM, and the cells were incubated for 72 h. Following 24 or 72 h drug exposure, 3-(4,5-dimethylthiazol-2-yl)-2,5-diphenyltetrazolium bromide (MTT) was added to the cells at a final concentration of 0.5 mg mL^−1^ and incubated for 2 h. Then, the culture medium was removed and the violet formazan (artificial chromogenic precipitate of the reduction of tetrazolium salts by dehydrogenases and reductases) dissolved in 200 μL DMSO. The optical density of each well (96-well plates) was quantified in triplicates at 550 nm using a multi-well plate reader, and the percentage of surviving cells was calculated from the ratio of absorbance of treated to untreated cells. The EC_50_ was calculated as the concentration reducing the proliferation of the cells by 50% and it is presented as a mean (± *SD*) of at least three independent experiments.

### Statistics

A minimum of three independent experiments were performed with the cells, with four replicates for each condition. The EC_50_ values were calculated, using GraphPad Prism, as the concentration reducing the viability of the cells or slices by 50%, relative to the untreated samples using a nonlinear fitting of log [compound concentration] vs. response and is presented as the mean ± *SD* of at least three independent experiments. Statistical testing was performed with two-way ANOVA with each individual experiment as random effect. In all graphs and tables the mean values and standard deviation (*SD*) are shown.

### ICP-MS studies

The concentrations of platinum, gold and copper were measured by inductively coupled plasma mass spectrometry (ICP-MS) using an Elan 6,000 spectrometer (Perkin Elmer Sciex, Concord, ON, Canada) and Micromist Nebulizer and a cyclonic spraychamber (Glas Expansion Pocasset, MA, U.S.). Nebulizer gas, lens voltage and RF power were optimized daily using a 10 μg/L Pt standard in 0.1% (V/V) HCl and 0.65% (V) HNO_3_, normal settings were: Rf power 1,350 W, nebuliser gas flow rate 0.95 L min^−1^, and lens voltgate 10 V. Acquisition parameters were 50 ms dwell time, 1 sweeps reading, 5 replicates and 25 readings per replicate, monitoring all or a subset of ^195^Pt^+^, ^196^Pt^+^, ^197^Au^+^, and ^63^Cu^+^. The instrument was tuned at the beginning of each analysis to ensure optimal operation.

Samples were dried by vacuum centrifugation (Eppendorf, concentrator plus) at 60°C for 2 h. Cell samples were digested using 200 μL 65% HNO_3_ and 50 μL 30% H_2_O_2_ overnight until the solution was clear. Samples were prepared for analysis by dilution to 5 mL with 0.65% (V/V) HNO_3_ and 0.1% (V/V) HCl. The external standards were prepared from a 10 mg/L Au and Pt stock solution (CPI International, Peak Performance, 4400-120213WG01, Lot# 12B214) or Cu stock solution (Plasma CAL, SCL SCIENCE, Q.C. no 4, Cat# 140-102-045, Lot SC5322198) with 0.65% (V/V) HNO_3_ and 0.1% (V/V) HCl. All reagents were of the highest available purity.

### Metal content determination after 24/72 h +/– inhibitor

A2780 or A2780cisR cells were plated into 25 cm^2^ culture flasks (CellStar, Grenier Bio, Germany) with RPMI 1640 medium up to a volume of 5 mL per flask. After 24 h, the cells were treated with a mixture of 15 or 20 μM cisplatin for 24 h, or 5 or 10 μM cisplatin for 72 h, +/– cimetidine or +/– CuCl_2_. Cimetidine was used at 300 μM concentration in H_2_O, and CuCl_2_ was used at 30 μM in H_2_O, both non-toxic to cancer cells. The same set-up was used for compound **1**, but the compound was incubated at 5 or 10 μM for 24 h, and 3 or 6 μM for 72 h, respectively. After 24 or 72 h, the medium was removed, and the cells were washed two times with 3 mL ice-cold PBS to remove dead cells. Thus, lysis buffer (500 μL) was added and the samples were stored on ice for 20 min. Afterwards, cells were detached by using a scrubber policemen and the cell suspension was transferred to a 1.5 mL Eppendorf tube. 200 μL of the sample were transferred to a new Eppendorf tube for ICP-MS analysis, while 5 μL of cell suspension was used for protein determination.

### Metal content determination during 120 min incubation +/– inhibitor

A2780 or A2780cisR cells were plated into 25 cm^2^ culture flasks (CellStar, Grenier Bio, Germany) with RPMI 1,640 medium up to a volume of 5 mL per flask. The next day, the cells were treated with 5 μM cisplatin or with 3 μM compound **1** (+/– cimetidine or +/– CuCl_2_) for 10′, 20′, 30′, 60′ ,and 120′ min. The samples were processed as described above.

### Protein assay

Protein determination was performed using BioRad DC^TM^ Protein Assay reagents A, B, and S (Bio-Rad). Using a 96-well-plate, 5 μL of standard (0, 5, 10, 15, 20, 25, and 30 mg/ml bovine serum albumin) and samples were analyzed in duplicate. First, 25.5 μL reagent-mix A+S (25 μL A + 0.5 μL S) was added and then 200 μL reagent B. The absorbance at 610 nm was measured after 15 min (stored in dark) using a PlateReader (Perkin Elmer, EnVision, 2104 Multilabel Reader).

### Fluorescence microscopy

A2780 cells were seeded (5 × 10^5^ for each sample) and grown on 8 well microscope plates, coated with Poly-L-lysine hydrobromide (Sigma-Aldrich, P6516) with RPMI medium. After 24 h, cells were incubated with various concentrations of Au(III) compound **1** in RPMI, without FCS for 1 h at 37°C. Afterwards, cells were rapidly washed with cold PBS and then fixed with 2% paraformaldehyde for 30 min at 4°C. For visualization of the nuclei with PI (propidium iodide), cells were permeabilized with 0.2% Triton X-100 for 20 min at 4°C and treated with 1 μg/μl of PI for 10 min at room temperature. Cells were washed once with PBS and then analyzed by confocal microscopy. As preparation for visualization, the plate wells were removed from the slide and glycerol was used to cover the slide with a glass cover slip. The fluorescence was analyzed using a Leica DM4000 B Automated Upright Microscope, equipped with the appropriate filters. PI was excited at 547 nm (emission wavelength 572 nm) and compound **1** at 358 nm (emission wavelength 461 nm, DAPI filter). The acquired images were obtained using the individual filters and a combined image, overlaying the fluorescence was acquired using the Leica microscope software.

## Results

### Synthesis and characterization

A novel cyclometallated Au(III) compound **1** was synthesized adapting already established procedures for similar complexes (Figure 2).(Bertrand et al., 2015) In details, the C^∧^N precursor [Au(py^b^-H)Cl_2_] (Cinellu et al., 1995) was reacted with the phosphine 3-[4-(diphenylphosphino)phenyl]-7-methoxy-2H-chromen-2-one (PPh_2_Arcoum) (Hanthorn et al., 2012) to yield the fluorescent Au(III) compound **1**. The complexation of the phosphine ligand with [Au(py^b^-H)Cl_2_] as well as the isomeric purity of compound **1** was analyzed by ^31^P(^1^H) NMR spectroscopy. The NMR spectrum (Figure S1a) shows a singlet at 31.5 ppm of the coordinated phosphorous shifted downfield by 35 ppm with respect to the corresponding precursor (Figure S1b) (Bertrand et al., 2015). The ^1^H NMR spectrum of **1** (Figure S2a) shows a downfield shift of the signal of the pyridine proton in position 6 by 0.05 ppm associated with a morphological change of the pyridine moiety. A splitting of the signals corresponding to the carbons of the two phenyl rings compared to the free ligand (Figure S2b) was observed due to their diastereotopic feature induced by the fixed geometry of the cyclometallated ligand as depicted in Figure S2c in the Supplementary Material. The same splitting of the signals of the two phenyl rings was observed in the ^13^C(^1^H) spectrum of **1** (Figure S3). Moreover, another splitting of the signal due to the coupling of the phosphorous atom with carbons and hydrogens is observed. Compound **1** in dichloromethane exhibits a typical absorption band at 361 nm and a fluorescence emission band of the coumarin chromophore at 429 nm (Figure S4). As previously observed, the complexation induces a bathochromic shift of the absorption wavelength with respect to free phosphine ligand (+ 33 nm), while there is no significant modification of emission wavelength (Dondaine et al., 2016). It is worth noting that complexation of gold(III) preserves the smart character of the coumarin-phosphine ligand that we highlighted for gold(I) complexes (Ali et al., 2015; Dondaine et al., 2016). Indeed, compound **1** displayed a quantum yield of fluorescence almost 8-fold higher than the free phosphine (38 and 5% respectively). This phenomenon will make possible to check the stability of the P-Au bond by microscopy of fluorescence experiment *in vitro*: if the bond breaks, the fluorescence emission will drop dramatically (Figure S5).

### Antiproliferative effects

The antiproliferative effects of the gold(III) compound **1** were studied in different human cancer cell lines in comparison to cisplatin, *via* a classical 3-(4,5-dimethylthiazol-2-yl)-2,5-diphenyl tetrazolium bromide (MTT) assay after 72 h incubation (see experimental for details). Specifically, the selected human cancer cell lines were A2780 and A2780cisR (ovarian carcinoma cisplatin sensitive and resistant, respectively), MCF7 (breast carcinoma), A549 (lung carcinoma) as well as against HCT116 p53 +/+ and –/– (colon carcinoma p53 expressing and knock-out, respectively). The tumor protein p53 is a tumor suppressor gene and known to be downregulated in cisplatin resistant cancer tissues (Li et al., 2007).

The obtained EC_50_ values are summarized in Table 1. Overall, compound **1** exhibits a moderate toxicity profile in all tested cell lines in line with previous studies on similar C^∧^N gold(III) complexes, while being more effective against the ovarian A2780 cancer cells (Bertrand et al., 2015). Compared to cisplatin, **1** shows a moderately higher toxic effect against A2780cisR, which suggests that different mechanisms of anticancer activity with respect to the Pt(II) agent may be in place, as well as possible alternative transport and accumulation pathways in this cell line. Instead, no statistically significant difference of EC_50_ values was observed in the HCT116 p53 –/– cells among the two drugs. This is different from what has been observed for other families of organometallic Au(I) complexes featuring N-heterocyclic carbene ligands (Estrada-Ortiz et al., 2017). Moreover, similar toxicity with respect to cisplatin was detected in the case of the non-cancerous HEK-293T cells. In addition, we also evaluated the I_max_, calculated as the survival rates at maximum inhibition, which provide a measure of drug maximal toxicity, while the EC_50_ values reflect the drug potency. As it can be seen from the I_max_ values reported in Table 1, compound **1** shows similar effects as cisplatin, made exception for the HCT116 p53 –/–, MCF7 and A549 cell lines, where the Pt(II) drug has a higher I_max_.

**Table 1:** Antiproliferative effects of gold(III) compound **1** (expressed as EC_50_ and I_max_ values) compared to cisplatin against different human cancer cell lines and non-tumorigenic HEK-293Tcells, after 72 h incubation.

		**EC**_**50**_ **(**μ**M)**^a^ **[I**_*****max*****_**(%)]**					
**Compound**	**A2780**	**A2780cisR**	**HCT116 p53** +**/**+	**HCT116 p53 –/–**	**MCF7**	**A549**	**HEK-293T**
1	2.4 ± 0.3 [0.044]	11 ± 0.5 [0.054]	9.8 ± 0.9 [0.058]	18.4 ± 1.1 [0.072]	12 ± 3 [0.03]	26± 5 [0.041]	10 ± 3 [0.030]
Cisplatin	2.3 ± 0.5 [0.065]	30 ± 1 [0.073]	5.3 ± 0.2 [0.056]	22.9 ± 2.3 [0.185]	20 ± 3 [0.124]	12 ± 1 [0.105]	8.6 ± 1.3 [0.030]

a *Data are expressed as mean ± SD (n ≥ 3)*.

Due to the highest activity against the ovarian cancer cells, both sensitive and resistant to cisplatin, these cells were selected for further experiments to investigate the transport mechanisms for the gold(III) compound **1** in comparison to cisplatin. Both A2780 and A2780cisR cells were recently evaluated from Sørensen et al. (2016) for the expression levels of CTR1, OCT2, and ATP7A/B transporters. Notably, the cisplatin resistant A2780cisR cells show a significantly higher expression of the copper efflux transporters ATP7A (1.3-fold) and ATP7B (5-fold) as well as of the cation uptake transporter OCT2 (1.3-fold). The copper uptake transporter CTR1 is on the other hand less expressed in A2780cisR cells compared to the A2780 (0.4-fold). These data suggest that the resistance observed in the A2780cisR cells is at least partly caused by a different balance between uptake and efflux of cisplatin resulting from a decreased influx *via* CTR1 and concomitant increased efflux of cisplatin by ATP7A/B, thereby decreasing the intracellular exposure of the cells to cisplatin.

### Studies on the mechanisms of transport

#### Competition experiments

The antiproliferative effects of compound **1** and cisplatin were determined after 24 and 72 h incubation in order to observe the development of the toxicity over time (Table 2). Overall, compound **1** is more potent in the A2780 cells, than in the resistant A2780cisR cells at both incubation times. In addition, after 24 h incubation, compound **1** is 5-fold more active than cisplatin in A2780 cells, and 7-fold more active in A2780cisR cells. However, after 72 h incubation, both compounds display the same cytoxicity in A2780, while the gold(III) compound is still ca. 3-fold more active in A2780cisR than cisplatin. In addition, there is no significant difference between the EC_50_ values at either 24 or 72 h treatment of A2780cisR cells with compound **1**. Instead, in A2780 cells, the gold(III) compound shows a 2-fold decrease of the EC_50_ values from 24 to 72 h incubation, indicating increase of activity during chronic exposure.

**Table 2:** EC_50_ values of compound **1** and cisplatin against A2780 and A2780cisR cells, incubated in absence and presence of CuCl_2_ or cimetidine (Cim), recorded after 24 and 72 h incubation.

	**EC**_**50**_ **(**μ**M)**^a^			
	**24 h**		**72 h**	
**Compound**	**A2780**	**A2780cisR**	**A2780**	**A2780cisR**
1	5.8 ± 1.5	15 ± 4	2.4 ± 0.3	11 ± 0.5
1 + Cim	6.6 ± 0.3	18.2 ± 6.1	0.7 ± 0.2^**^	8.1 ± 0.5^*^
1 + CuCl_2_	4.1 ± 0.3	6.1 ± 1.9^*^	0.2 ± 0.1^**^	3.1 ± 1.0^**^
Cisplatin	26 ± 2	103 ± 3	2.3 ± 0.5	30 ± 1
Cisplatin + Cim	22.0 ± 2.1	91.7 ± 4.2	1.0 ± 0.1^**^	21.3 ± 1.1^**^
Cisplatin+ CuCl_2_	22.2 ± 1.3	51.0 ± 4.9^**^	0.5 ± 0.2^**^	18.0 ± 0.6^**^

a *Data are expressed as mean ± SD (n ≥ 3)*.

*The reported values are the mean ± SD of three independent experiments. For statistical analysis, the two way ANOVA was used*.

* *p ≤ 0.05*,

** *p ≤ 0.01 indicate the difference is significant when compared to samples treated with the metallodrugs only (control)*.

In order to evaluate the involvement of OCT2 and CTR1 uptake transporters in the metallodrugs accumulation, the compounds' antiproliferative effects were further tested in the presence of transporter inhibitors or competitor substrates, respectively (see experimental section for details). If these transporters are involved in the drug uptake, their inhibition should lead to a reduced intracellular accumulation of the metal complexes and to a decrease in cytotoxic effects, and thus, an increase in EC_50_ values. Conversely, inhibiting possible efflux transporters such as MATE and ATP7A/B should lead to metallodrug accumulation and enhancement of its antiproliferative effects. Cimetidine (300 μM, Cim) was selected as inhibitor for OCTs and MATE, and CuCl_2_ (30 μM) as competitive substrate of both CTR1 and ATP7A/B (Spreckelmeyer et al., 2014). Experiments were performed in A2780 and A2780cisR cells after 24 and 72 h incubation with the compounds. It should be noted that CuCl_2_ and cimetidine do not show any anticancer effect on A2780 or A2780cisR cell lines at the selected concentrations (data not shown). The obtained results are summarized in Table 2 and in Figures S6, S7.

After 24 h incubation, neither cimetidine nor CuCl_2_ showed an effect on the potency of gold(III) compound **1** or cisplatin in A2780 cells (Figure S6A). However, in A2780cisR cells (Figure S6B), both cisplatin and compound **1** had a significantly increased activity when co-incubated with CuCl_2_. For cisplatin the resistance was only partly reduced, but for gold(III) compound **1** the resistance was fully compensated resulting in a similar toxicity in both cell lines. These results support the hypothesis that CuCl_2_ may interfere with the metallodrugs de-toxification mechanisms involving the copper efflux transporters (ATP7A/B), which are expressed at much higher levels in the A2780cisR cells than in the wild type A2780 cells (Sørensen et al., 2016). Apparently, these efflux transporters play a minor role in the wild type A2780 cells. Furthermore, the lack of effect of cimetidine on the efficacy of both cisplatin or compound **1** (Figure S6A) in both cell lines might indicate that drug uptake processes by OCTs and efflux by MATE are not relevant.

After 72 h incubation of the A2780 cells, both cimetidine and CuCl_2_ increased the potency of compound **1** significantly, 3.5-fold and 12-fold, respectively. For cisplatin, a 2.5-fold and 4.5-fold increased toxicity could also be observed with cimetidine and CuCl_2_, respectively (Figure S7A). The same effect was observed in A2780cisR cells, but to a lower extent (Figure S7B). This discrepancy may be rationalized by the hypothesis that for both compounds, at 72 h incubation, efflux mechanisms are inhibited by CuCl_2_ and cimetidine, possibly via inhibition of ATP7A/B and MATE, respectively. However, it should be noted that in conditions of chronic drug exposure other pathways may cause the observed increased cytotoxic effects other than transport mechanisms.

### Metal accumulation studies by ICP-MS

After evaluating the toxic effects of both compounds in cancer cells in the presence and absence of transport inhibitors/competitors, the metal content (Au or Pt) was determined by inductively coupled plasma mass spectrometry (ICP-MS) to gain further insights into the drug accumulation mechanisms. For these experiments two concentrations of the metal compounds were chosen based on their EC_50_ values at the selected incubation time (24 or 72 h), namely EC_50_ or 2-fold EC_50_ concentrations against the A2780 cells were considered for the two drugs. The obtained results are reported in Table 3 and expressed in ng metal/μg protein.

**Table 3:** Metal content determination by ICP-MS after exposure of A2780 and A2780cisR cells with compound 1 and cisplatin, incubated in absence and presence of CuCl_2_ (30 μM) or cimetidine (Cim, 300 μM), recorded after 24 and 72 h incubation.

	**Au and Pt content [ng metal/**μ**g protein]**			
	**24 h**		**72 h**	
**Treatment**	**A2780**	**A2780cisR**	**A2780**	**A2780cisR**
**COMPOUND 1**				
[5 μM]	0.04 ± 0.01	0.04 ± 0.01	nd	nd
[10 μM]	0.12 ± 0.03	0.10 ± 0.02	nd	nd
[5 μM] + CuCl_2_	0.08 ± 0.02	0.08 ± 0.01	nd	nd
[10 μM] + CuCl_2_	0.48 ± 0.06	0.21 ± 0.01	nd	nd
[5 μM] + Cim	0.04 ± 0.01	0.04 ± 0.01	nd	nd
[10 μM] + Cim	0.26 ± 0.06	0.12 ± 0.02	nd	nd
[3 μM]	nd	nd	0.08 ± 0.01	0.05 ± 0.01
[6 μM]	nd	nd	0.19 ± 0.02	0.15 ± 0.01
[3 μM] + CuCl_2_	nd	nd	0.16 ± 0.03	0.12 ± 0.01
[6 μM] + CuCl_2_	nd	nd	0.15 ± 0.01	0.28 ± 0.03
[3 μM] + Cim	nd	nd	0.09 ± 0.02	0.06 ± 0.02
[6 μM] + Cim	nd	nd	0.25 ± 0.05	0.16 ± 0.01
[15 μM]	0.07 ± 0.01	0.06 ± 0.01	nd	nd
[20 μM]	0.17 ± 0.04	0.08 ± 0.02	nd	nd
[15 μM] + CuCl_2_	0.09 ± 0.01	0.07 ± 0.01	nd	nd
[20 μM] + CuCl_2_	0.20 ± 0.03	0.13 ± 0.01	nd	nd
[15 μM] + Cim	0.08 ± 0.02	0.06 ± 0.01	nd	nd
[20 μM] + Cim	0.25 ± 0.02	0.10 ± 0.01	nd	nd
[5 μM]	nd	nd	0.04 ± 0.02	0.02 ± 0.01
[10 μM]	nd	nd	0.07 ± 0.02	0.05 ± 0.01
[5 μM] + CuCl_2_	nd	nd	0.05 ± 0.02	0.02 ± 0.01
[10 μM] + CuCl_2_	nd	nd	0.09 ± 0.02	0.07 ± 0.01
[5 μM] + Cim	nd	nd	0.02 ± 0.01	0.01 ± 0.01
[10 μM] + Cim	nd	nd	0.05 ± 0.02	0.04 ± 0.01

*The reported values are the mean ± SD of three independent determinations. nd, not determined*.

#### Au accumulation in cancer cells

**Figure 3:**
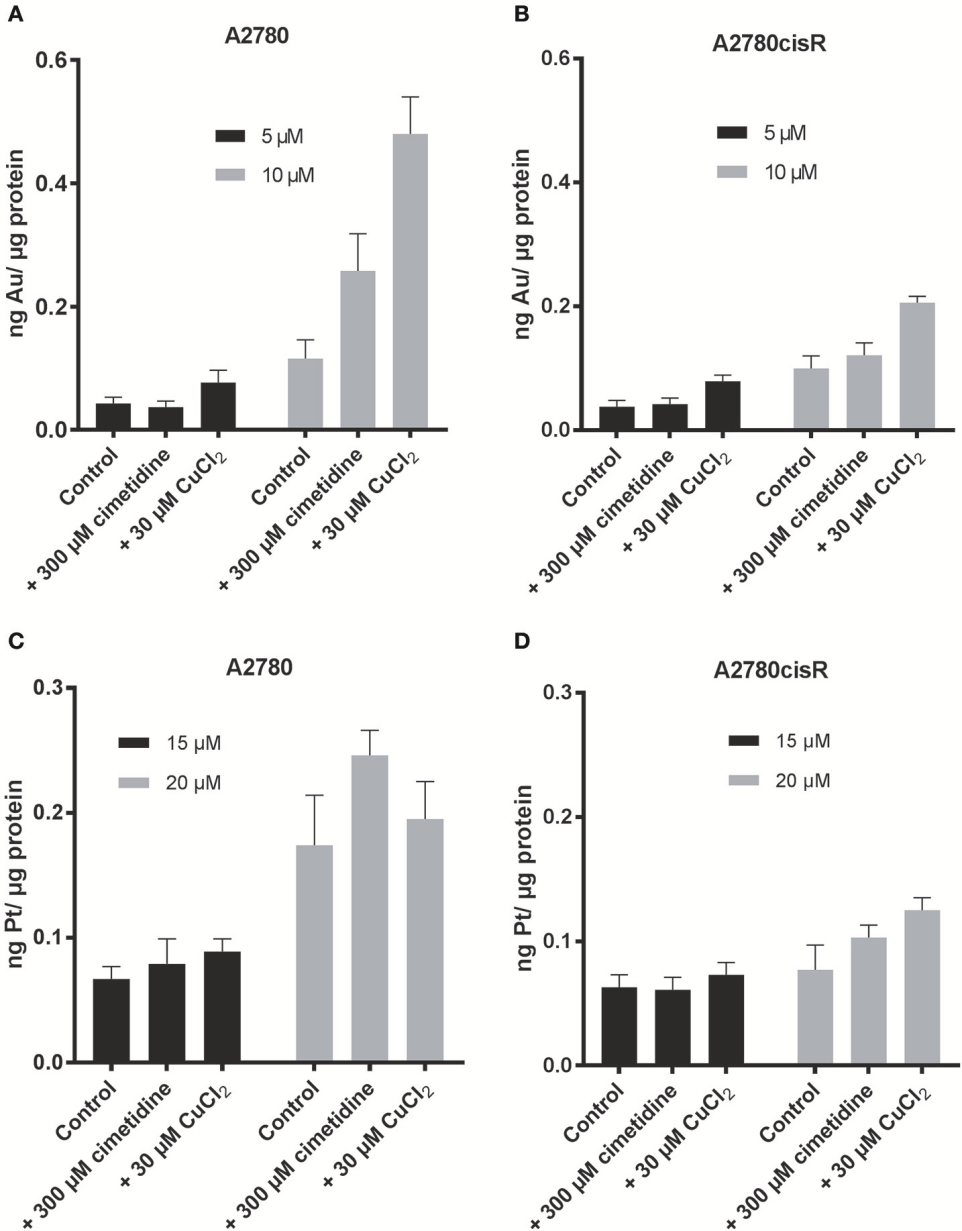
Au content after 24 h incubation of **(A)** A2780 **(B)** A2780cisR cells treated with 5 μM (black) and 10 μM (gray) of compound **1**. Pt content after 24 h incubation of **(C)** A2780 and **(D)** A2780cisR with 15 μM (black) and 20 μM (gray) cisplatin. Data are expressed as mean ± *SD* (*n* = 3).

When cells were treated with compound **1**, as expected, the metal content increased as a function of concentration (Table 3, Figure 3, Figure S8). Interestingly, no significant differences in Au content could be observed between the two cell lines (sensitive or resistant to cisplatin) treated with the same concentrations of the gold(III) compound **1** at either incubation times. Considering the above mentioned marked differences in EC_50_ values for 1 between the two cell lines and the different incubation times (Table 2), the differences in accumulation mechanisms may not play a major role in determining the overall antiproliferative effects of the gold(III) complex.

When cells were co-treated for 24 h with the gold compound and CuCl_2_, increased accumulation of Au was observed in both cell lines treated with 10 μM concentration of compound **1** (Table 3, Figure 3). The effect of the CuCl_2_ co-incubation was most marked in the A2780 cells with a 4-fold increase in Au content compared to the controls, whereas the metal content increased only of 2-fold in the A2780cisR. A 2-fold increase was observed at 5 μM in both cell lines, but this increase was not statistically significant. Overall, these results are in line with the enhanced effect of the copper/drug co-incubation on the observed antiproliferative effects (Table 2).

Co-incubation of the gold(III) compound for 24 h with 300 μM cimetidine resulted in a slightly increased accumulation of Au only in the A2780 wild type cells at 10 μM of compound **1**, but not at 5 μM, and no effect was seen in the A2780cisR cells (Figure 3) in line with the antiproliferative activity data (Table 2).

The same type of experiments was repeated after 72 h treatment of cells with compound **1** at either 3 or 6 μM. After 72 h incubation in the presence of CuCl_2_, a ca. 2-fold increase in Au content was observed both in A2780 cells and in A2780cisR cells at either metallodrug concentrations, except for the co-incubation of 6 μM **1** in the cisplatin sensitive cells, where no difference was observed with respect to controls (Table 3, Figure S8). This may be explained by the fact that after 72 h, 6 μM is about 2-fold the EC_50_ value for **1** in A2780 cells, which may have caused extensive cell death, altering membrane integrity and Au leakage from the cells. Concerning the cimetidine treatment, this did not have any significant effect on the Au content of the cells after 72 h of incubation (Table 3, Figure S8).

### Pt accumulation in cancer cells

Afterwards, the Pt content was evaluated in A2780 and A2780cisR cells treated with cisplatin after 24 (Table 3, Figure 3) or 72 h incubation (Table 3, Figure S9) by ICP-MS. At 24 h, the Pt content was the same for both cell lines, despite the known differences in the expression of hCTR1 and OCT transporters (Sørensen et al., 2016). The increase in metal content was concentration dependent over 24 h in the A2780 cells, while a similar trend could not be observed in the cisplatin resistant cells, where the Pt content remained substantially unaltered at both drug concentrations and incubation times (Figure 3, Figure S9). This concentration dependent effect could not be observed in the case of the A2780cisR cells also after 72 h incubation, supporting the idea that higher detoxification and possibly efflux mechanisms may be in place. Notably, no significant difference in Pt content could be observed after co-incubation with either cimetidine or CuCl_2_. Thus, these data cannot explain the observed increase in anticancer effects (Table 2).

#### Copper accumulation in cancer cells

The viability results of cisplatin treated human ovarian cancer cells showed a decreased EC_50_ value after co-incubation with CuCl_2_. However, the ICP-MS data did not show an increased Pt content that could be responsible for such an effect. In order to reinforce the evidence that inhibition of the copper transporters by cisplatin or by the Au(III) complex is not relevant to their mechanism of anticancer effects, we investigated whether the Cu^2+^ content was increased due to the co-incubation with each metallodrug. Concerning copper uptake, CTR1 affinity to Cu is between 0.6 μM in fibroblasts and 13 μM in murine hepatocytes, (Lee et al., 2002) while ATP7B binding affinity to Cu is 2.5 ^*^ 10^−17^ M (Hilário-Souza et al., 2011). Both transporters are not only selective for Cu, but also for example for Ag(I), Cd(II) and Fe(III) (Liang et al., 2014).

**Figure 4:**
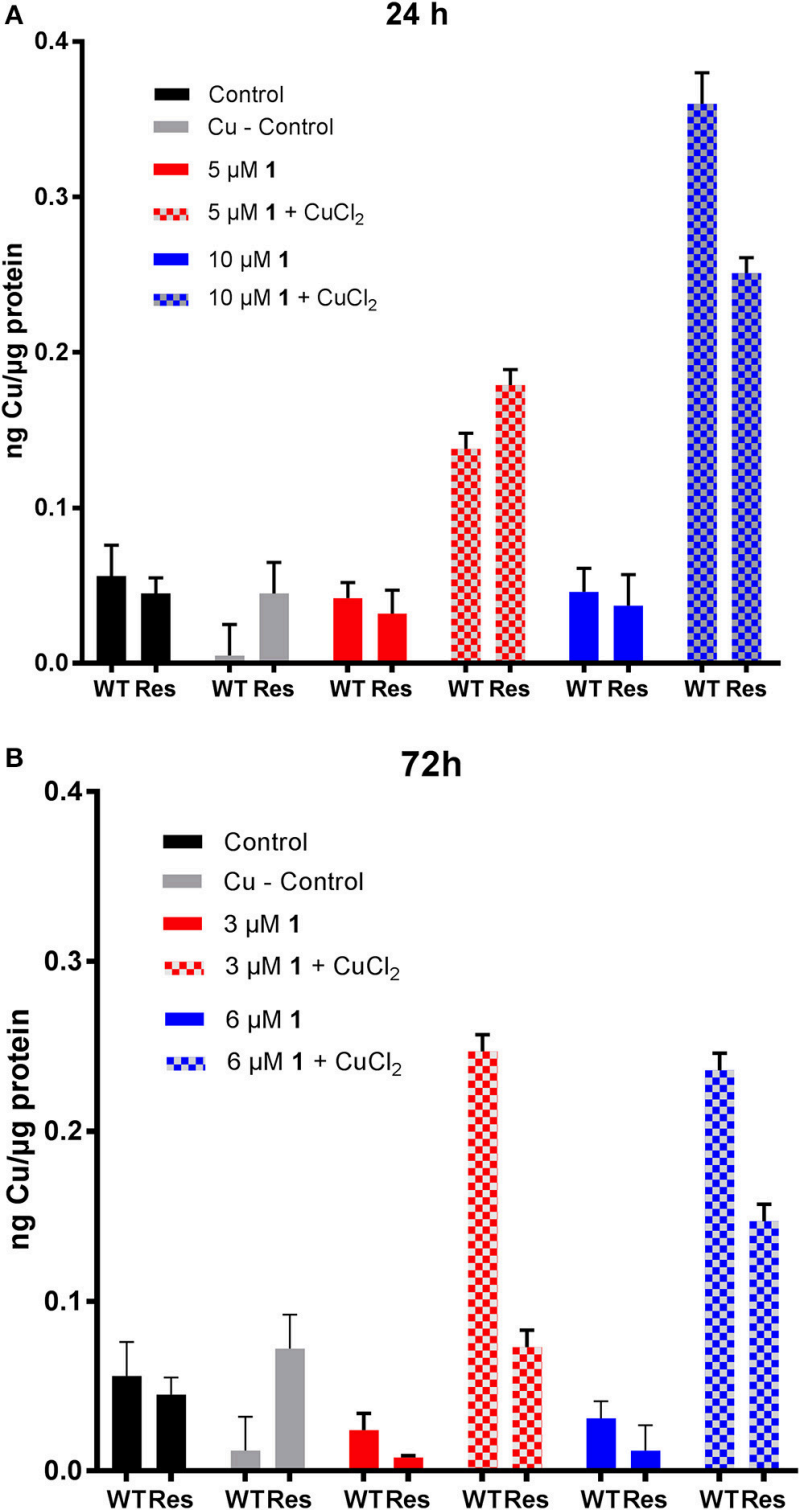
Evaluation of copper content in human ovarian cancer cells treated with **1**. Cu content in A2780 (WT) and A2780cisR (Res) cells after **(A)** 24 h and **(B)** 72 h incubation with 30 μM CuCl_2_ and different concentrations of compound **1**. Data are expressed as mean ± *SD* (*n* = 3).

Initially, copper content was evaluated in both the wild-type A2780 and cisplatin resistant A2780cisR cells by ICP-MS since the different CTR1/ATP7A/B transporter expression levels(Sørensen et al., 2016) may have an effect in its accumulation. The obtained results show that both cell lines have the same basal copper content (0.056 ± 0.02 ng Cu in A2780 and 0.045 ± 0.01 ng Cu in A2780cisR). Furthermore, treatment with CuCl_2_ (30 μM) did not significantly alter these basal levels (Figure 4).

Concerning metallodrug treated cells, it could be observed that the copper content did not decrease in the cell lines upon treatment with the Au(III) compound **1** for 24 (5 and 10 μM) and 72 h (3 and 6 μM), at any selected drug concentration (Figure 4). Instead, co-incubation of 1 with CuCl_2_ induced an increased copper accumulation compared to controls after 24 h in both cell lines. At 72 h, the Cu content was markedly increased mainly in the A2780 cells. Overall, the ICP-MS data showed an increased Au and Cu content after 24 and 72 h incubation in the CuCl_2_ co-incubation experiments, which may be responsible for the observed increased antiproliferative effects in the same cells (Table 3). The effect of the increased Cu-content is not fully understood yet, but an additive or even synergic anticancer effect with Au may be possible.

**Figure 5:**
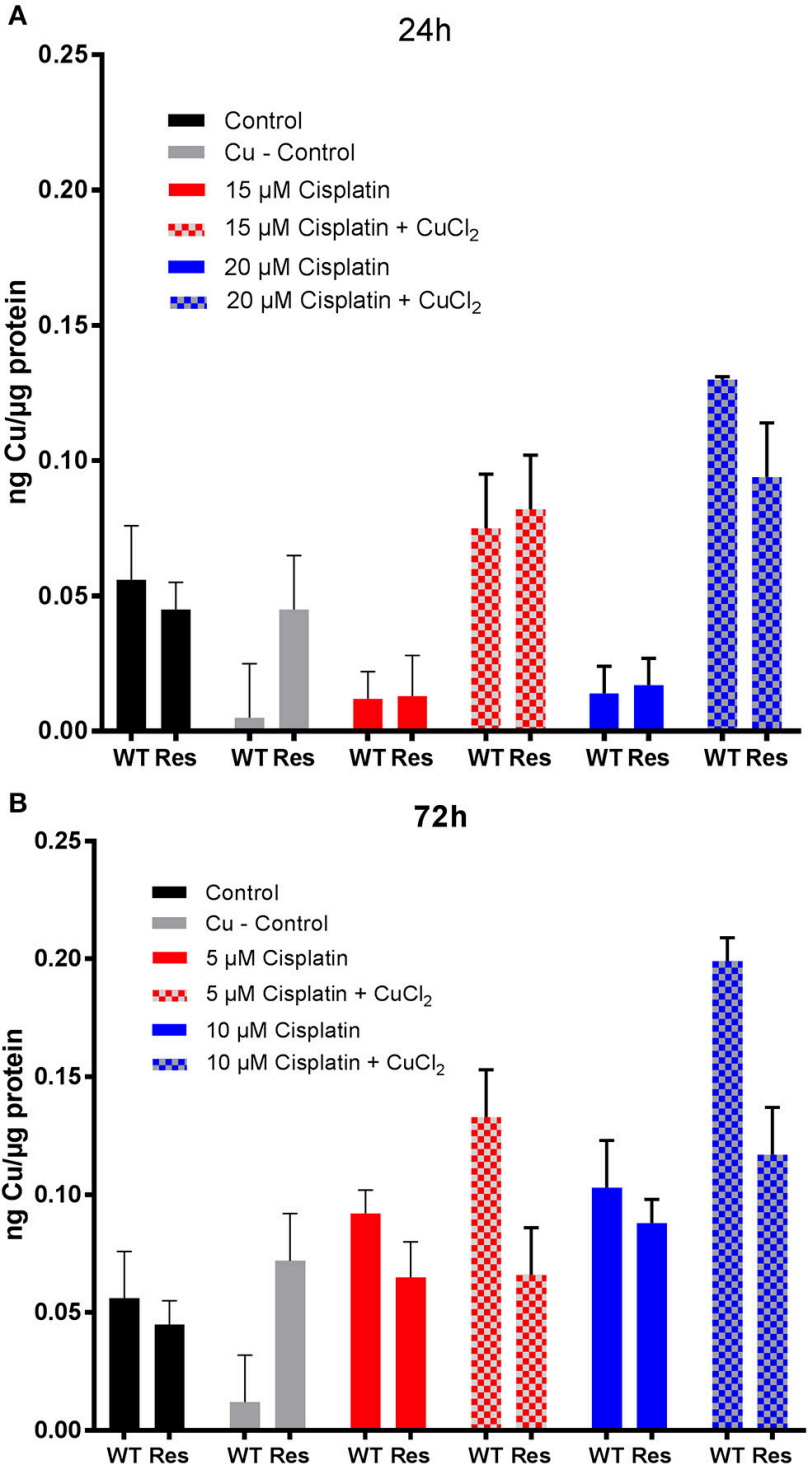
Evaluation of copper content in human ovarian cancer cells treated with cisplatin. Cu content in A2780 (WT) and A2780cisR (Res) cells after **(A)** 24 h and **(B)** 72 h incubation with 30 μM CuCl_2_ and different concentrations of cisplatin. Data are expressed as mean ± *SD* (*n* = 3).

Considering cisplatin treatment, no significant difference in the Cu content between the controls and cisplatin treated cells could be observed in both cell lines after 24 and 72 h incubation (Figure 5). Instead, after 24 h, the Cu content significantly increases at both concentrations of cisplatin co-incubated with CuCl_2_ in both cell lines. However, this effect was absent in the A2780cisR cells incubated for 72 h. The ICP-MS data showed no increased Pt and Cu content after 24 h and 72 h incubation in the CuCl_2_ co-incubation experiments (Table 3).

To summarize the results concerning the gold(III) complex, the following can be concluded: after 24 h, co-incubation of **1** with CuCl_2_, but not with cimetidine, induced a significantly higher antiproliferative effects in A2780cisR cells, whereas in A2780 cells no effect was seen of either CuCl_2_ or cimetidine. After 72 h incubation, co-incubation of **1** with CuCl_2_ resulted in an increase in cytotoxicity and increase in Au and Cu content in both cell lines as demonstrated by ICP-MS. Based on these results two explanations can be suggested for these effects: (i) compound **1** is a substrate for ATP7A/B, and inhibition of the efflux transporter ATP7A/B by excess CuCl_2_ results in a higher Au accumulation and higher drug potency, and/or (ii) the increased overall cytotoxicity is a result of additive or synergistic effects of Au and Cu accumulation via different transport mechanisms. Furthermore, a direct involvement of the CTR1 or OCT2 in the uptake of the gold drug, as well as of MATE in its efflux, could not be supported based on our studies. The increased efficacy of compound **1** in the presence of cimetidine after 72 h of incubation in A2780 cells, but not after 24 h, is difficult to explain, as no concomitant increased accumulation of Au was observed by ICP-MS. Studies with other, more selective, inhibitors for each single drug transporter are needed to confirm these results.

Concerning cisplatin, similarly to compound **1**, 24 h drug incubation with CuCl_2_ shows a significant decrease in viability in A2780cisR cells, although not as strong as for the gold(III) compound **1**. After 72 h, co-incubation of cisplatin with either cimetidine or CuCl_2_ leads to an increase in cytotoxicity, but no increase in Pt content could be observed in both cell lines as demonstrated by ICP-MS. This result, together with the evidence for increased Cu content in A2780 cells, leads to the hypothesis that copper accumulation is the reason for the observed enhanced anticancer effects in this cell line.

#### Metal uptake studies

**Figure 6:**
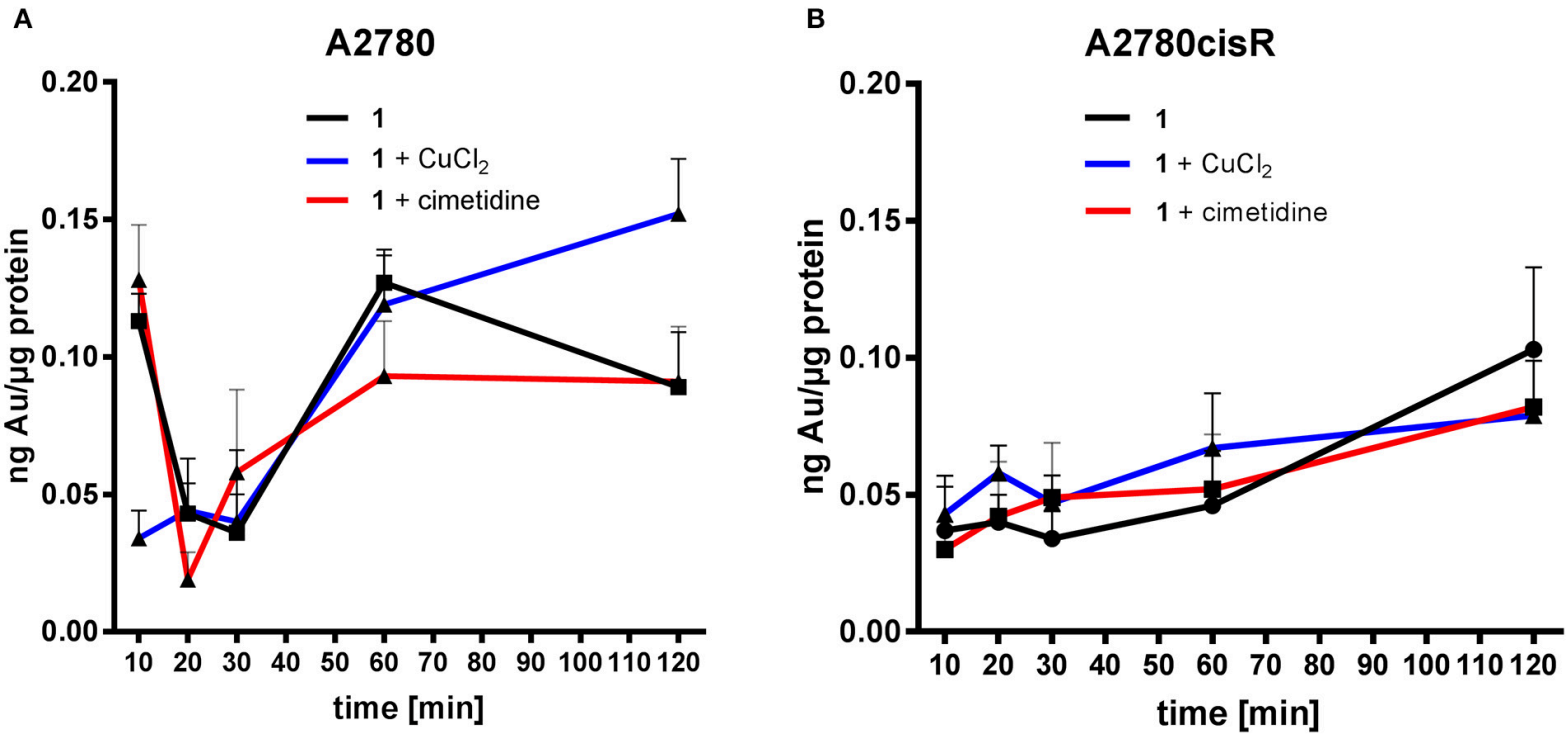
Gold uptake determination in cancer cells–Au content [ng metal/μg protein] in A2780 **(A)** and A2780cisR cells **(B)** treated with **1** and co-treatment with cimetidine or CuCl_2_. Data are expressed as mean ± *SD* (*n* = 3).

**Figure 7:**
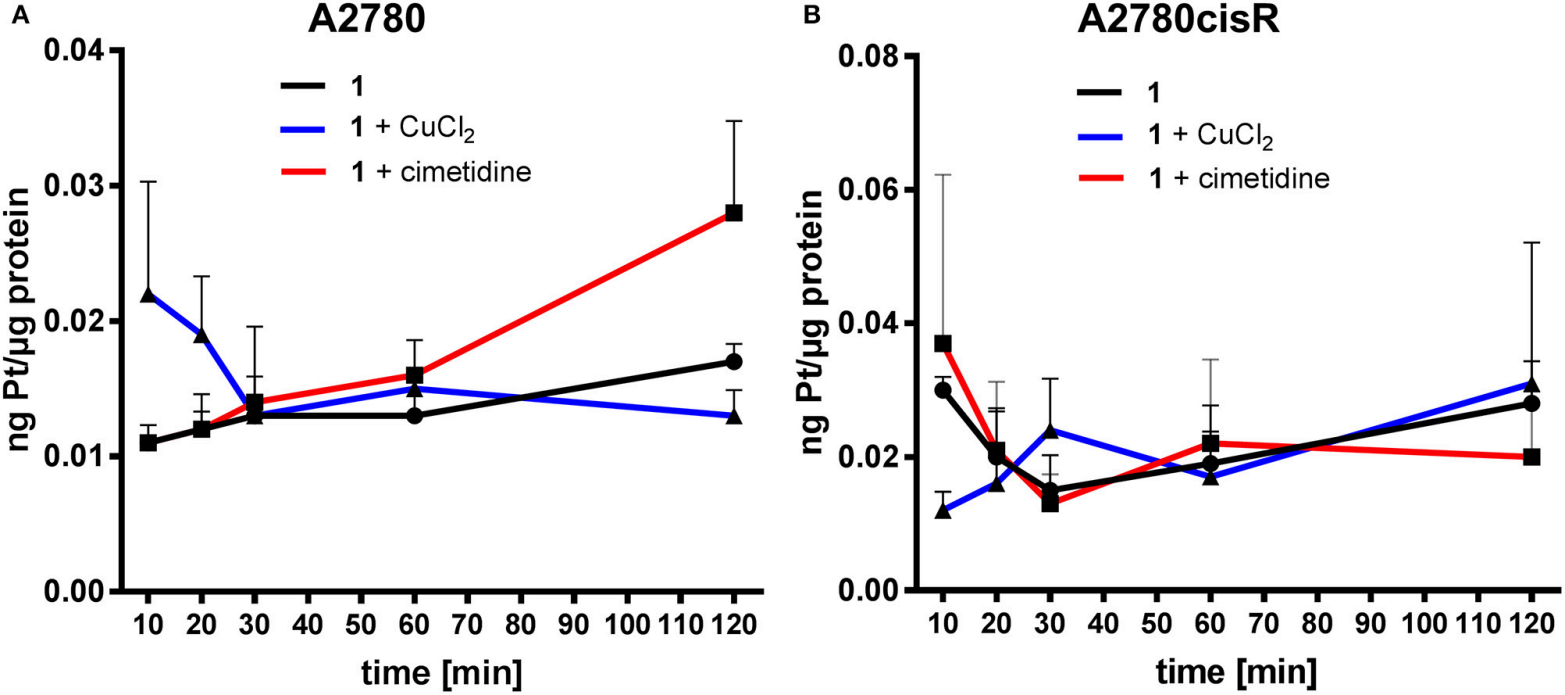
Platinum uptake determination in cancer cells–Pt content [ng metal/μg protein] in A2780 **(A)** and A2780cisR cells **(B)** treated with cisplatin and co-treatment with cimetidine or CuCl_2_. Data are expressed as mean ± *SD* (*n* = 3).

In order to specifically evaluate uptake mechanisms of metallodrugs in cancer cells, we measured the metal content (Au, Pt) in both A2780 and A2780cisR at different time points during the first 120 min of incubation in cells by ICP-MS. Thus, samples were taken after 10, 20, 30, 60, and 120 min and the obtained results are reported in (Figures 6, 7).

In the case of compound **1**, co-treatment with cimetidine did not affect the Au uptake in both cell lines at any recorded concentration. When cells were co-incubated with CuCl_2_, a significantly higher Au content could be observed only after 120 min in the A2780 cells (Figure 6A), but not in the cisplatin resistant ones (Figure 6B). Overall, both cimetidine and CuCl_2_ do not change the uptake of compound **1**. It should be noted that, between 10 and 20 min incubation, a drop in Au content can be observed in A2780 cells. We do not have an explanation for that thus far, but it should be further investigated in additional experiments.

In the case of cisplatin, the Pt content in cells co-incubated with cimetidine or CuCl_2_ did not show any difference over 120 min in both cell lines (Figure 7). A similar trend was observed in the co-treatment with cimetidine, made exception for the significantly increased Pt content recorded at 120 min in A2780 cells. Considering the whole set of data, it is also worth noting that metal accumulation is more efficient in the case of the gold(III) compound **1** compared to cisplatin in both cancer cell lines over time (see summary of the obtained data in Table 3).

In line with the aforementioned accumulation studies, a direct involvement of OCTs or CTR1 in the uptake of cisplatin was not observed. Otherwise, inhibition by cimetidine should have resulted in lower cytotoxicity and lower Pt, accumulation in the cells, which instead was not recorded. It should be also considered that Holzer and Howell showed that, as a consequence of cisplatin and copper incubation, the CTR1 transporter undergoes rapid down-regulation in human ovarian 2008 cancer cells, and that it might be internalized from the membrane, potentially leading to a decreased cisplatin uptake (Holzer and Howell, 2006). However, a decreased Pt uptake was also never evidenced in our results. Similarly, a direct involvement of MATE or ATP7A/B in the accumulation of cisplatin could not be confirmed.

### Fluorescence microscopy studies

**Figure 8:**
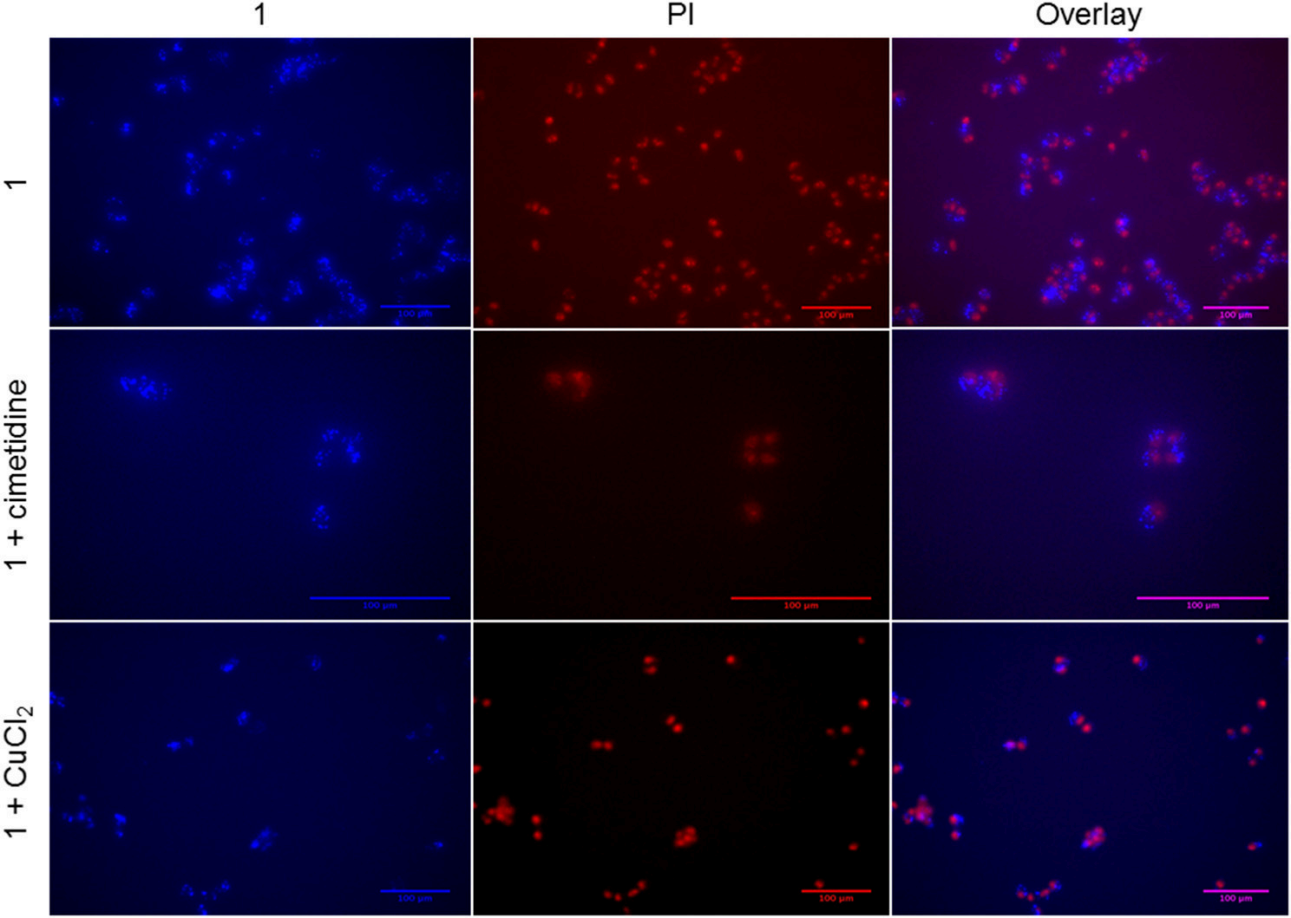
Fluorescence microscopy of A2780 cells treated for 2 h with compound **1** (5 μM) with/without 300 μM cimetidine or with/without 30 μM CuCl_2_.

To investigate the sub-cellular localization of the Au complex, we performed fluorescence microscopy experiments on A2780 cells treated with Au(III) compound **1** and the two inhibitors cimetidine and CuCl_2_. Propidium iodide (PI) staining was used to evidence cell nuclei. The pictures show the A2780 cells after 2 h incubation with gold(III) compound **1** (2-fold the EC_50_) (Figure 8). The strong fluorescence observed indicates that the coumarin-phosphine ligand is still coordinated to gold(III) cation. The compound itself does not co-localize with PI in the nuclei, but clearly enters the cells and is localized in organelles in the cytoplasm. For example, a similar distribution pattern was observed for a gold(I) complex featuring the same coumarin ligand as **1** (Ali et al., 2015). In this case, the gold(I) complex was hypothesized to accumulate in lipid rafts, cellular domains containing plasma membrane proteins and lipids, which are involved in the regulation of numerous cell functions, including signaling, trafficking, migration, adhesion, and growth. This observation points toward a different mechanism of action compared to cisplatin and suggests that the DNA damage may not play a pivotal role in the toxicity of the Au compound. Co-incubation with either cimetidine or CuCl_2_ did not change the intracellular accumulation pattern (Figure 8).

## Conclusions

In the past years, several studies have been carried out to elucidate the mechanisms of uptake and efflux of cisplatin in cancer cells and kidney slices, as well as in numerous *in vitro* models of cancer cells (Spreckelmeyer et al., 2014). Despite the numerous research efforts, such transport mechanisms are not yet fully understood. In addition, the mechanisms leading to activity/toxicity and cellular accumulation of new experimental anticancer gold complexes have received scarce attention, albeit the numerous compounds' structures published in the literature. Recently, we have reported on a promising family of gold(III) C^∧^N complexes as cytotoxic agents, with [Au(py^b^-H)PTACl] (py^b^-H = C^∧^N cyclometallated 2-benzylpyridine)(Bertrand et al., 2015) (Figure 1) as one of the most effective derivatives in cancer cells *in vitro*. In order to broaden the scope of our work and develop a compound trackable in cells via fluorescence microscopy, we report here on the cytotoxic effects of a new fluorescent gold(III) C^∧^N complex **1** studied in a panel of human cancer cell lines in comparison to cisplatin *in vitro*. The results show that **1** is more active than cisplatin in a number of cell lines, including MCF7, HCT16 p53 –/–, and A2780cisR cells. However, it has similar antiproliferative effects for other cancer cells, including the ovarian A2780 cells. The compound is also moderately cytotoxic toward the non-tumorigenic HEK cells. Additionally, we evaluated for the first time the involvement of different uptake and efflux transporters—namely OCTs, CTR1, MATEs, and ATP7A/B—in the accumulation mechanisms of **1** in human ovarian cancer cells *in vitro*, using competitive inhibitors/substrates and determining the intracellular metal content by ICP-MS. The results are compared with those obtained for cisplatin in the same experiments.

The uptake and intracellular accumulation of the gold(III) compound **1** is more efficient than that of cisplatin, as judged from the metal content assessed in both A2780 and A2780cisR cells by ICP-MS. In general, while uptake transporter OCT2 and CTR1 are unlikely to be involved in the transport mechanisms of both compounds in the selected cell lines, as well as in their antiproliferative effects, the efflux transporters ATP7A/B should be investigated further in the case of the gold complex. The preliminary fluorescence microscopy studies indicate that the gold compound accumulates intracellularly mainly in cytoplasmic organelles other than nuclei. Further studies are necessary to establish if these corresponds to mitochondria or lysosomes among others.

Overall, these initial results may constitute the basis for future more extensive studies on experimental metallodrugs transports and their relevance to the mechanism of activity/resistance toward different types of cancer, highlighting the differences with classical platinum-based chemotherapeutics.

## Data availability statement

All relevant data is contained within the manuscript: All datasets [GENERATED/ANALYZED] for this study are included in the manuscript and the Supplementary Material.

## Author contributions

AC, SaS, and StS contributed conception and design of the study. EB, BB, and AC contributed to the design, synthesis and characterization of the gold compound. SaS performed the anticancer studies *in vitro* and the data interpretation. SaS and MvdZ performed the ICP MS experiments and the statistical analysis. AC and SaS wrote the first draft of the manuscript, while StS, EB, and BB wrote sections of the manuscript. All authors contributed to manuscript revision, read and approved the submitted version.

### Conflict of interest statement

The authors declare that the research was conducted in the absence of any commercial or financial relationships that could be construed as a potential conflict of interest.
