# Supplementary material for: Relevance of Copper and Organic Cation Transporters in the Activity and Transport Mechanisms of an Anticancer Cyclometallated Gold(III) Compound in Comparison to Cisplatin
Source: Front Chem. 2018 Sep 4;6:377. doi: 10.3389/fchem.2018.00377 (PMC6131305; doi:10.3389/fchem.2018.00377)
Supplement: Supplementary file 1 [file Table_1.DOCX]

Supplementary Material

Relevance of copper and organic cation transporters in the activity and transport mechanisms of an anticancer cyclometallated gold(III) compound in comparison to cisplatin

Sarah Spreckelmeyer^1,2^, Margot van der Zee^1^, Benoît Bertrand^1,3^, Ewen Bodio^3^, Stefan Stürup^4^ and Angela Casini^1,5,*^

*** Correspondence:** Corresponding Author: [casinia@cardiff.ac.uk](mailto:casinia@cardiff.ac.uk)

#
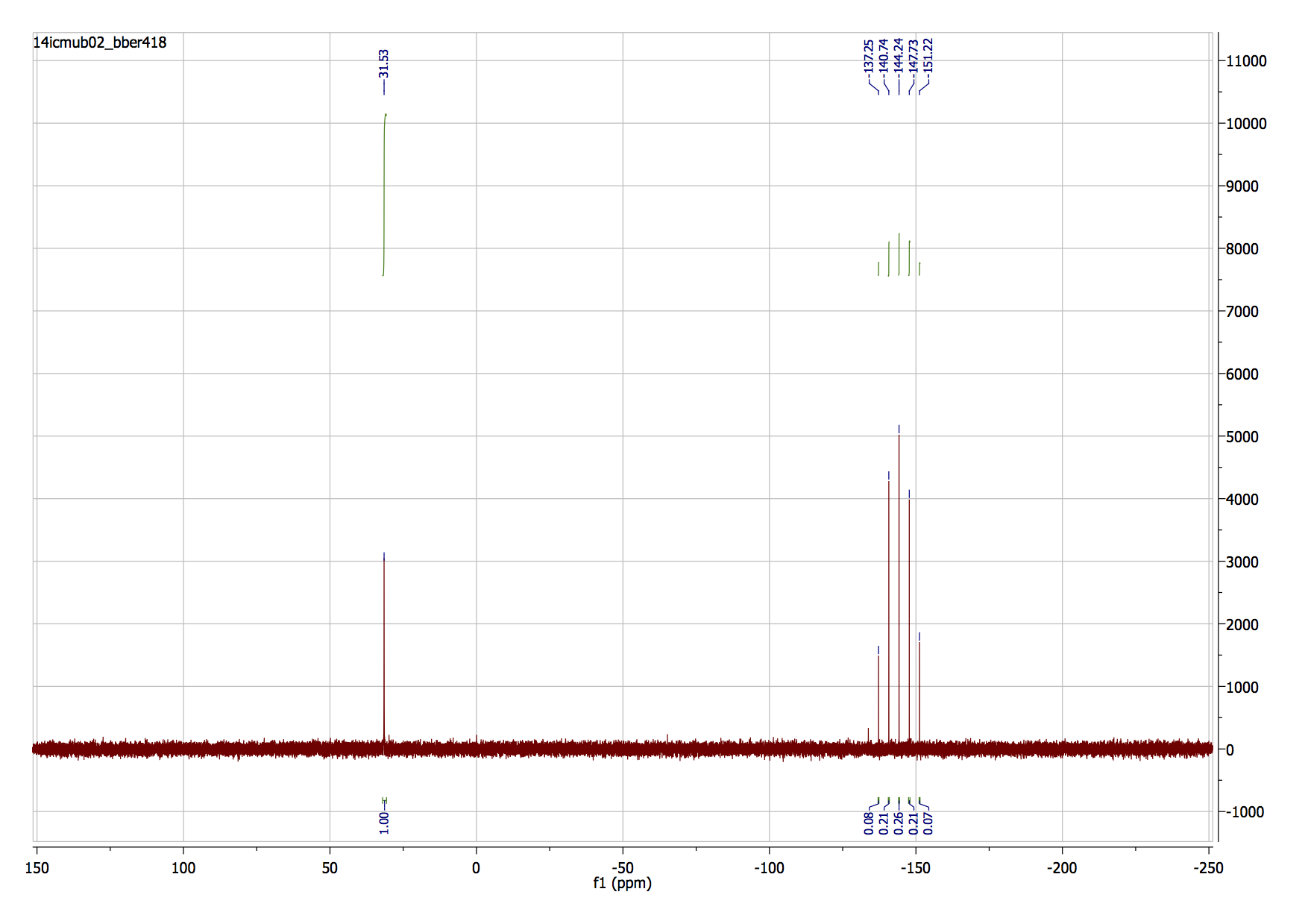


# Figure S1a. ^31^P{^1^H} NMR of 1 in acetone d_6_.


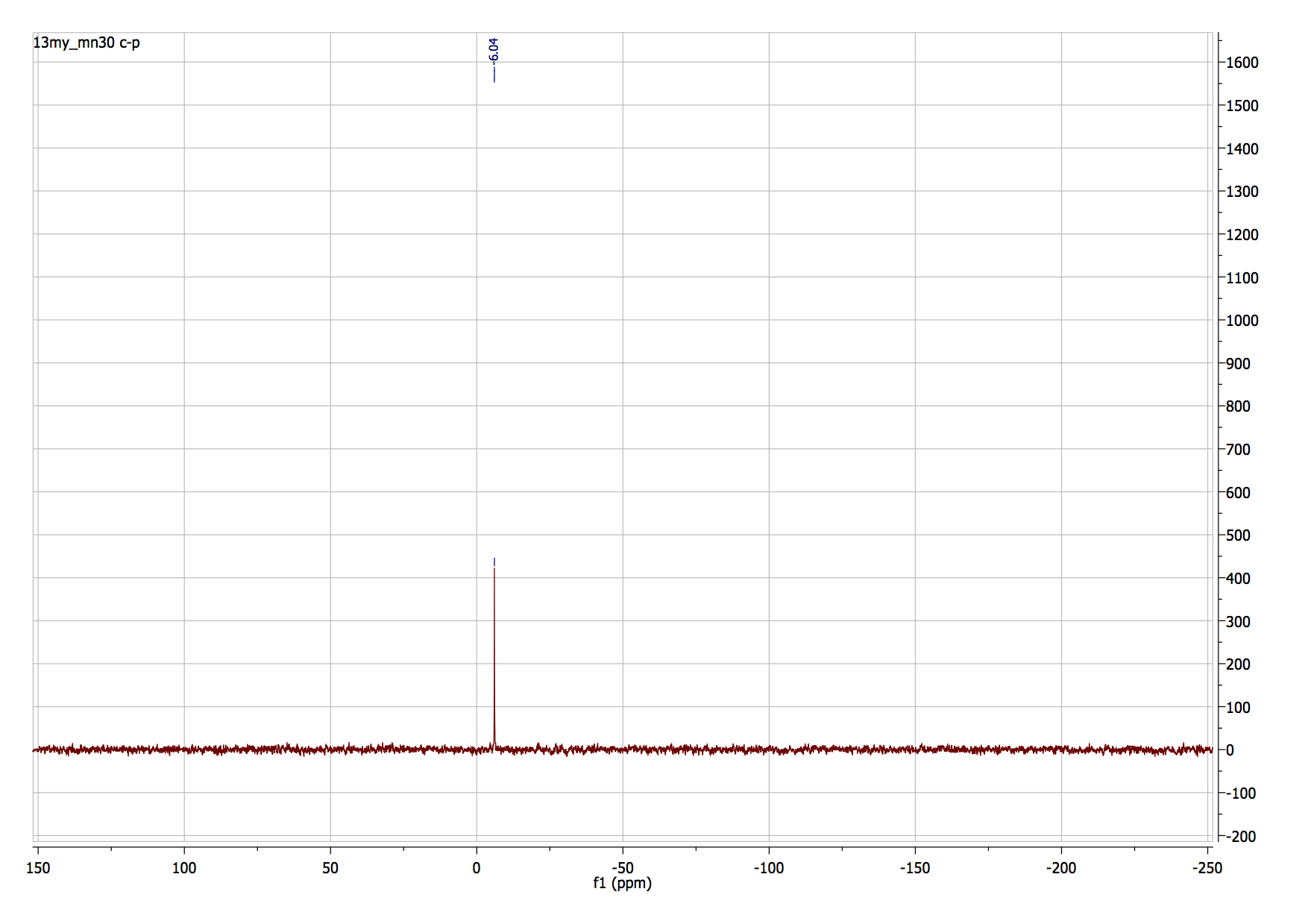


Figure S1b. ^31^P{^1^H} NMR of ligand PPh_2_coum in acetone-d_6_.


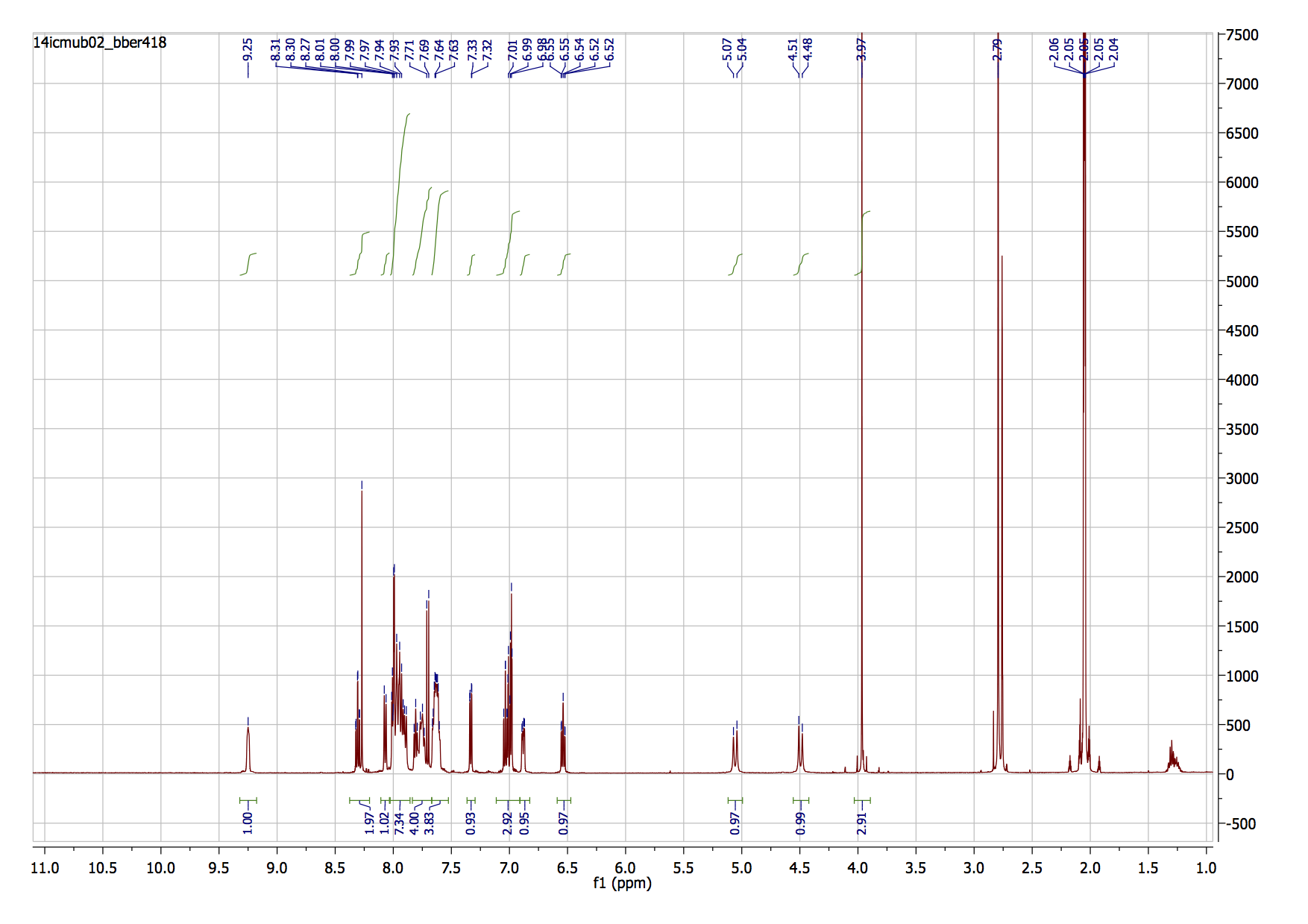


Figure S2a. ^1^H NMR of 1 in acetone d_6_.


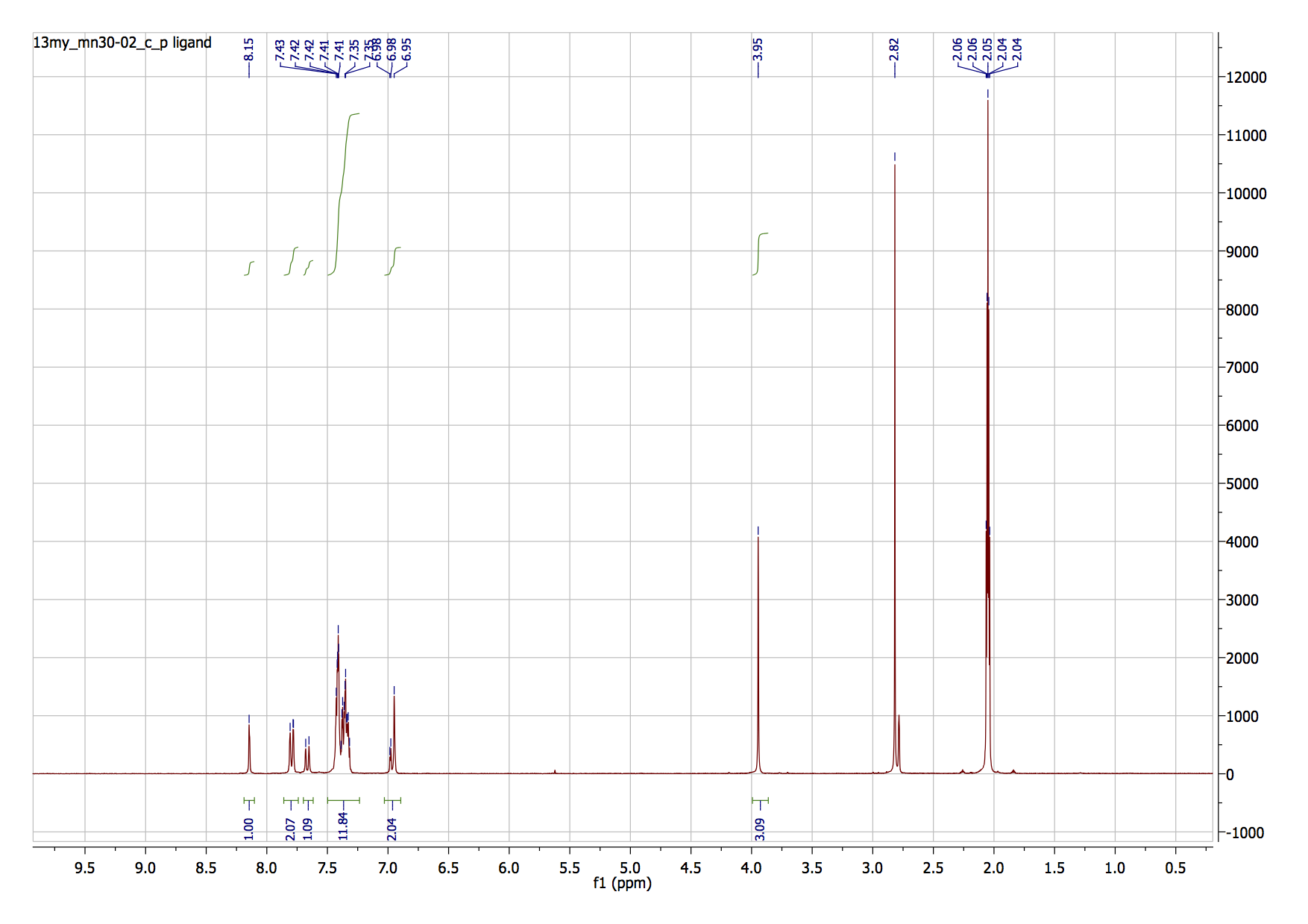


Figure S2b. ^1^H NMR of ligand PPh_2_coum in acetone d_6_.

**Figure S2c. General scheme of the two possible boat-like stereoisomers of [Au(py^b^-H)PCl] (1).**


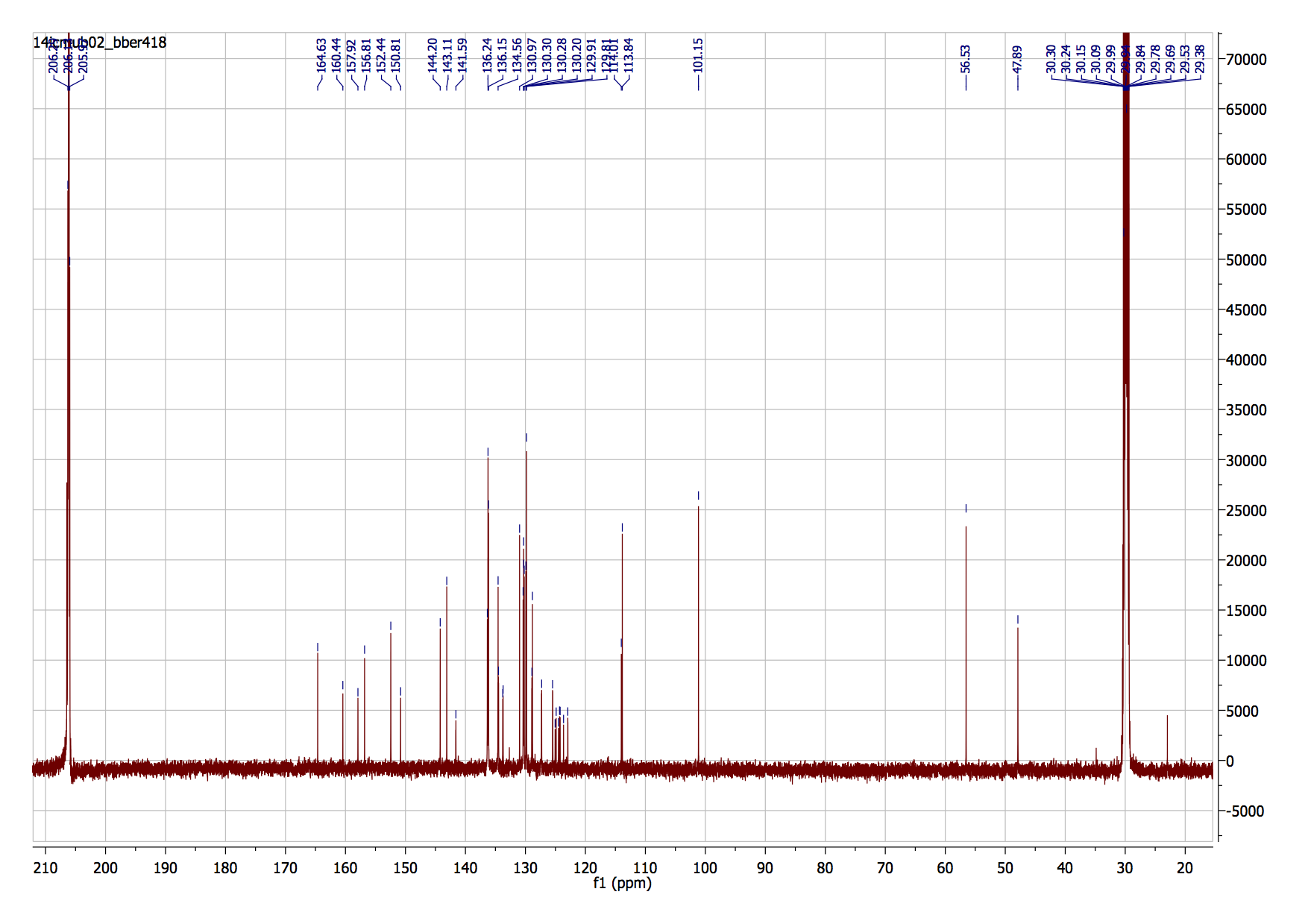


Figure S3. ^13^C{^1^H} NMR of 1 in acetone d_6_.

Figure S4. Absorption, excitation and emission spectra of the gold(III) compound 1 (dichloromethane, 2.5 x 10^-5^ M).


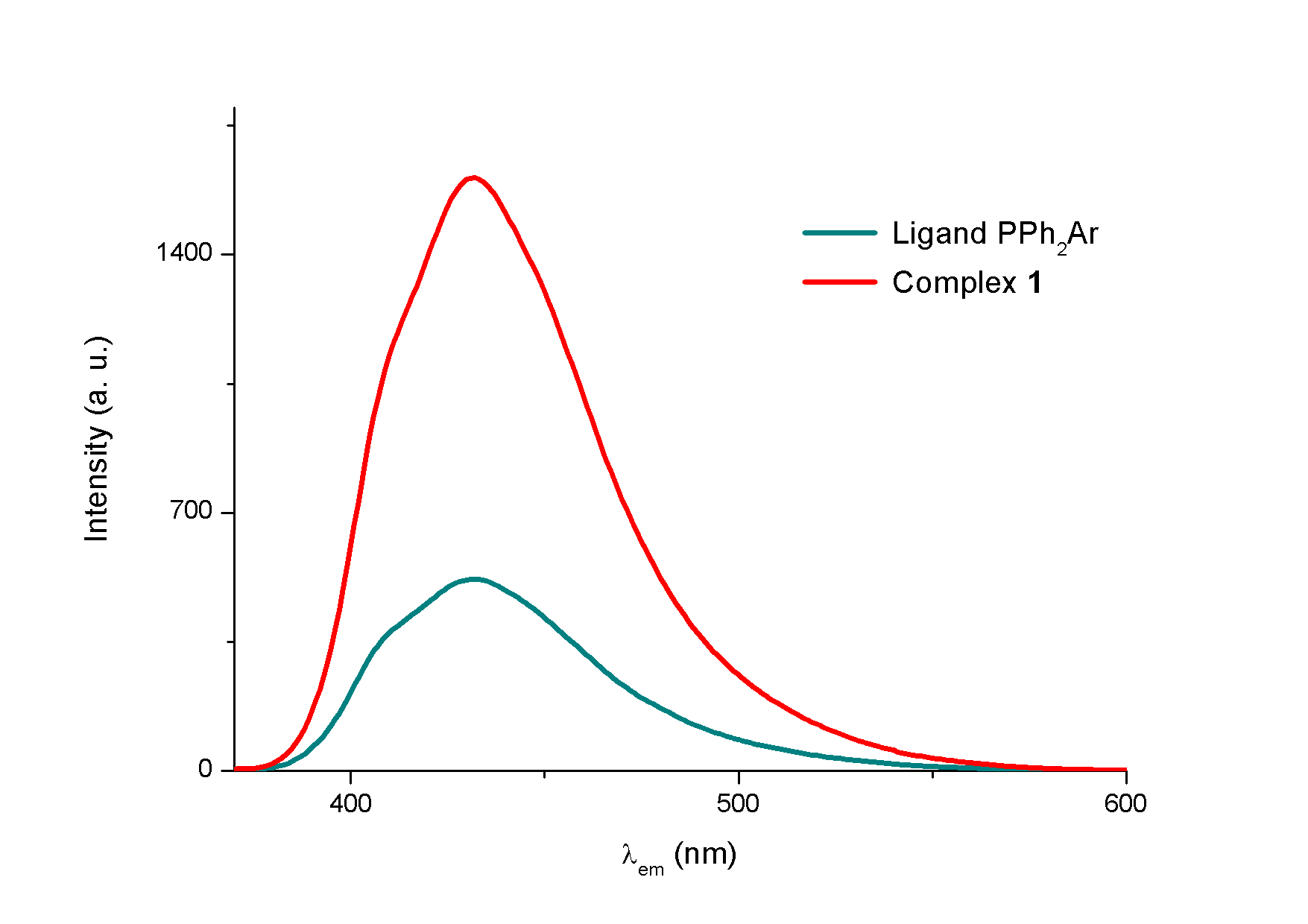


Figure S5. Comparison of the emission spectra of complex 1 and the free ligand PPh2Ar at a concentration of 2.5 10^-5^ M in dichloromethane at 298 K.


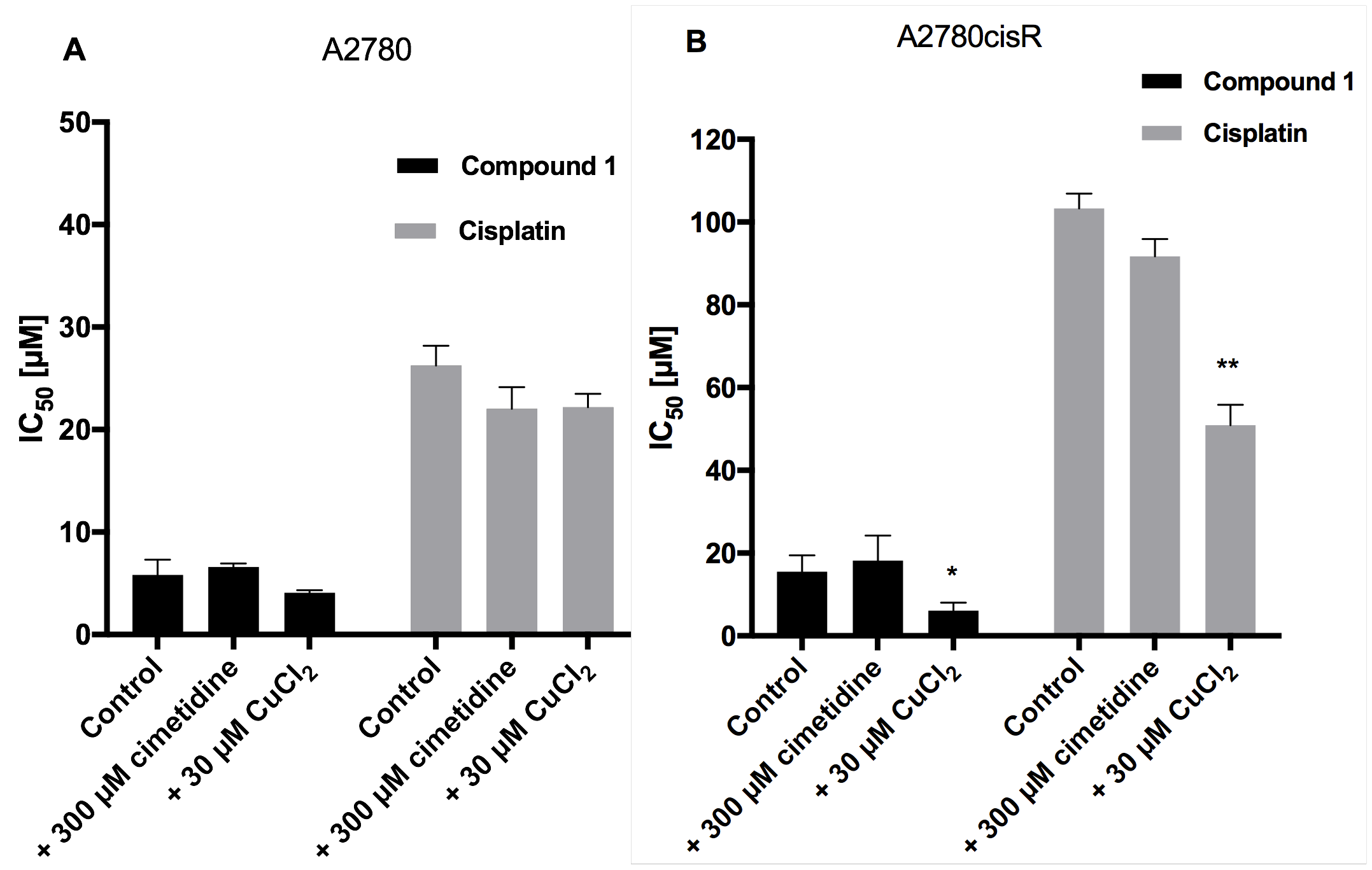


Figure S6. EC_50_ values of compound 1 and cisplatin on (A) A2780 and (B) A2780cisR after 24 h incubation. Data are expressed as mean ± SD (n≥3). For statistical analysis, the Two-way ANOVA was used. * (p ≤ 0.05), ** (p ≤ 0.01) indicate the difference is significant when compared to samples treated with the metallodrugs only (control).


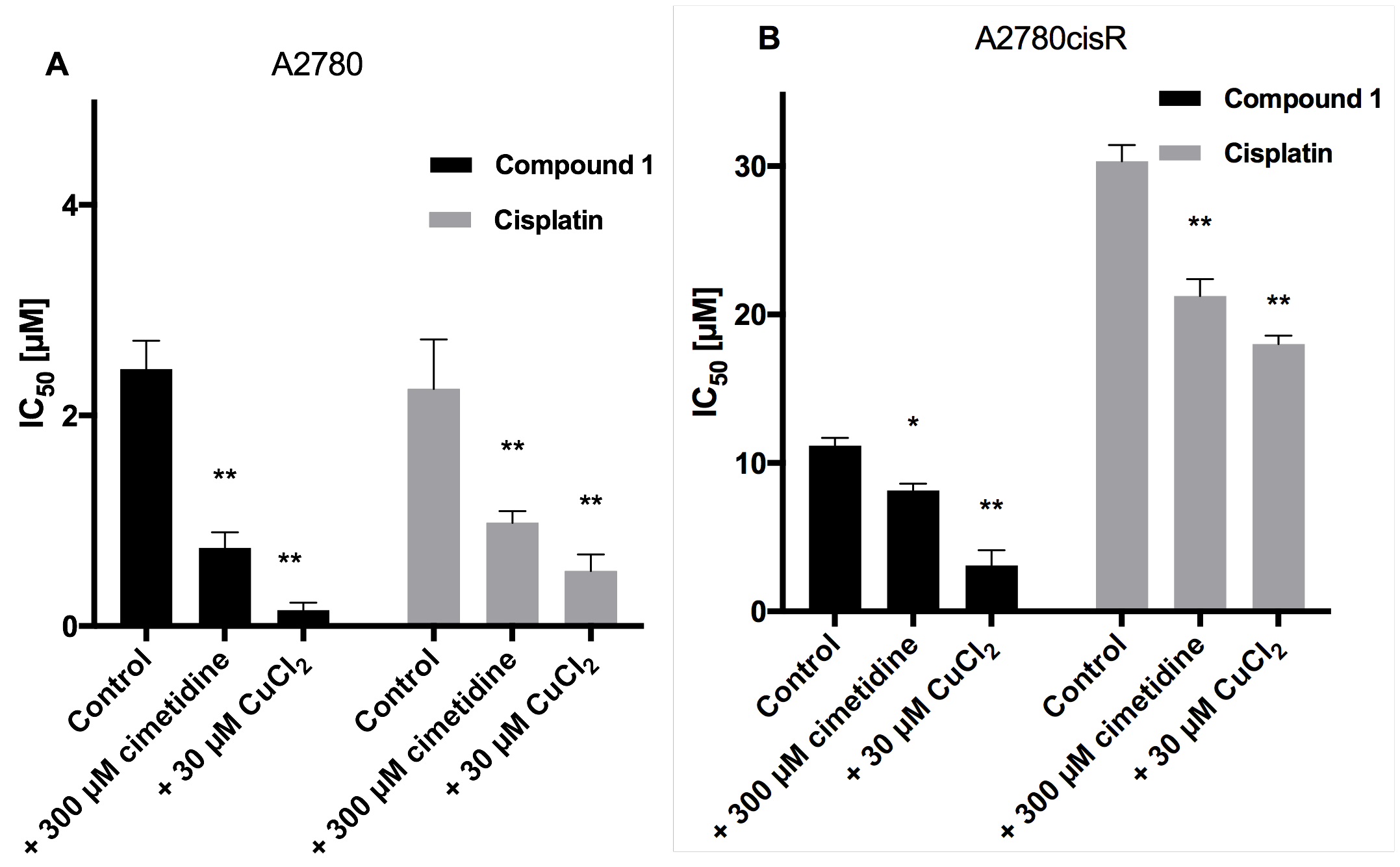


Figure S7. EC_50_ values of compound 1 (black) and cisplatin (grey) on (A) A2780 and (B) A2780cisR after 72h incubation. Data are expressed as mean ± SD (n≥3). For statistical analysis (Two-way ANOVA) * (p ≤ 0.05), ** (p ≤ 0.01) indicate the difference is significant when compared to the metallodrugs treated samples (control).


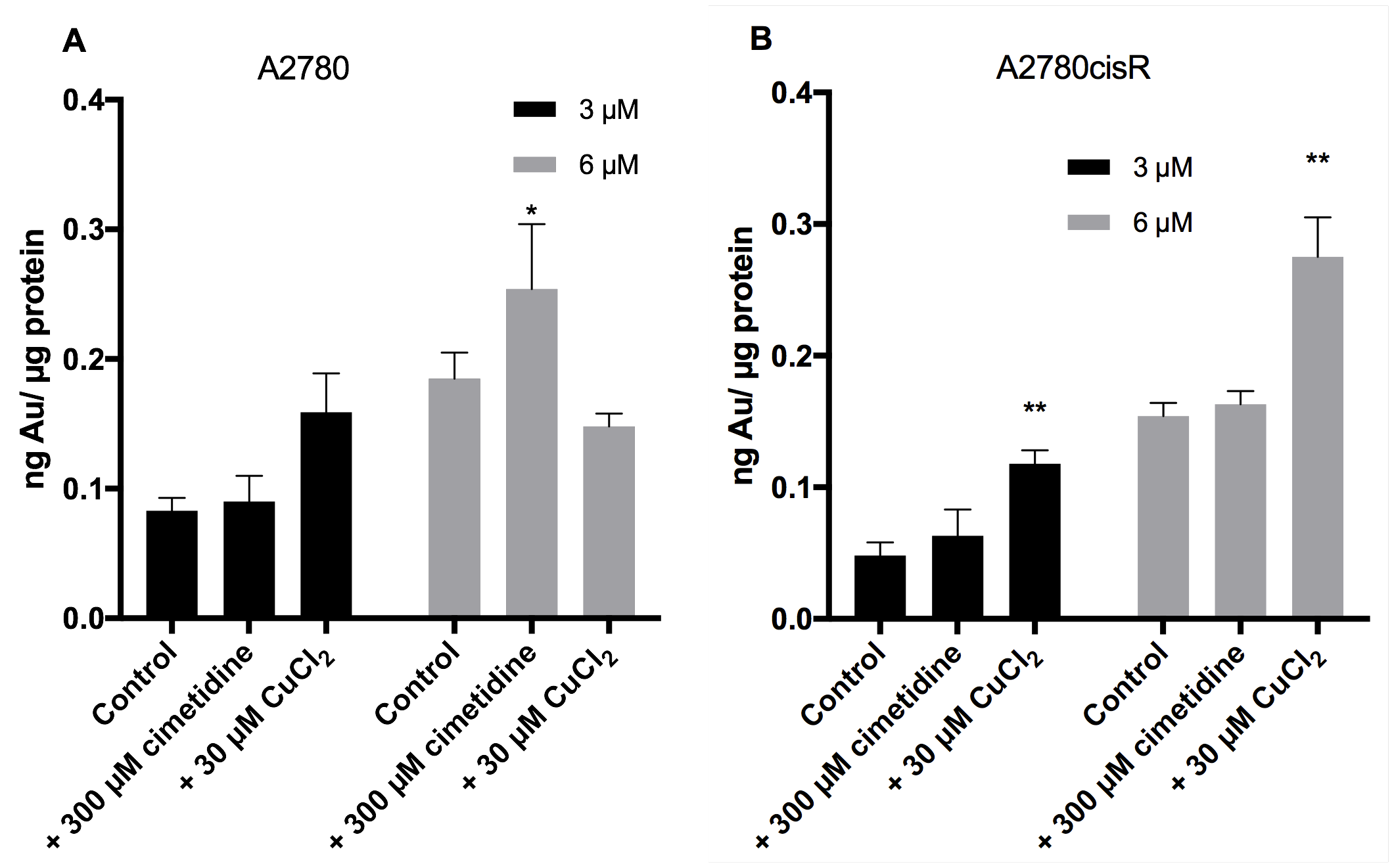


Figure S8. Au content after 72 h incubation of (A) A2780 and (B) A2780cisR cells treated with 3 µM (black) and 6 µM (grey) of compound 1. Data are expressed as mean ± SD (n=3).


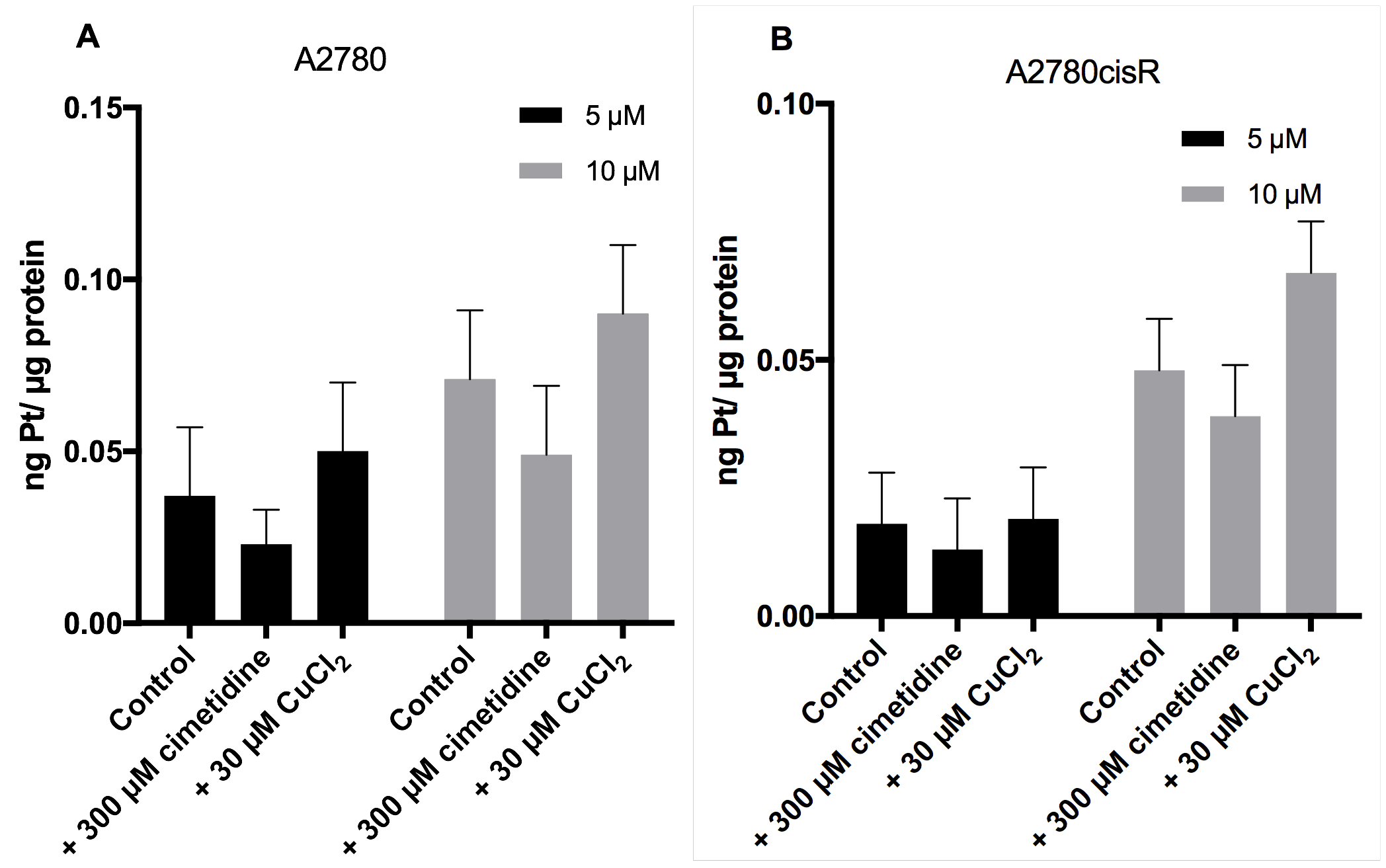


Figure S9. Pt content after 72 h incubation in (A) A2780 (B) A2780cisR cells, treated with 5 µM (black) and 10 µM (grey) cisplatin. Data are expressed as mean ± SD (n=3).
